# Altered Protein Networks and Cellular Pathways in Severe West Nile Disease in Mice

**DOI:** 10.1371/journal.pone.0068318

**Published:** 2013-07-10

**Authors:** Christophe Fraisier, Luc Camoin, Stéphanie Lim, Mahfoud Bakli, Maya Belghazi, Patrick Fourquet, Samuel Granjeaud, Ab D. M. E. Osterhaus, Penelope Koraka, Byron Martina, Lionel Almeras

**Affiliations:** 1 Unité de Parasitologie, URMITE UM 63, Institut de Recherche Biomédicale des Armées (Armed Forces Biomedical Research Institute, IRBA), Marseille, France; 2 Inserm, U1068, CNRS, UMR7258, CRCM, Aix-Marseille Univ, UM 105, Marseille, France; 3 Marseille Protéomique, Institut Paoli-Calmettes, Marseille, France; 4 Department of Virology, Erasmus MC, Rotterdam, The Netherlands; 5 Aix-Marseille Université, CNRS, UMR 7286, Marseille, France; 6 Aix-Marseille Université, Unité de Recherche en Maladies Infectieuses et Tropicales Emergentes (URMITE), UM 63, CNRS 7278, IRD 198, Inserm 1095, WHO Collaborative Center for Rickettsioses and Other Arthropod-borne Bacterial Diseases, Faculté de Médecine, Marseille, France; Blood Systems Research Institute, United States of America

## Abstract

**Background:**

The recent West Nile virus (WNV) outbreaks in developed countries, including Europe and the United States, have been associated with significantly higher neuropathology incidence and mortality rate than previously documented. The changing epidemiology, the constant risk of (re-)emergence of more virulent WNV strains, and the lack of effective human antiviral therapy or vaccines makes understanding the pathogenesis of severe disease a priority. Thus, to gain insight into the pathophysiological processes in severe WNV infection, a kinetic analysis of protein expression profiles in the brain of WNV-infected mice was conducted using samples prior to and after the onset of clinical symptoms.

**Methodology/Principal Findings:**

To this end, 2D-DIGE and gel-free iTRAQ labeling approaches were combined, followed by protein identification by mass spectrometry. Using these quantitative proteomic approaches, a set of 148 proteins with modified abundance was identified. The bioinformatics analysis (Ingenuity Pathway Analysis) of each protein dataset originating from the different time-point comparisons revealed that four major functions were altered during the course of WNV-infection in mouse brain tissue: i) modification of cytoskeleton maintenance associated with virus circulation; ii) deregulation of the protein ubiquitination pathway; iii) modulation of the inflammatory response; and iv) alteration of neurological development and neuronal cell death. The differential regulation of selected host protein candidates as being representative of these biological processes were validated by western blotting using an original fluorescence-based method.

**Conclusion/Significance:**

This study provides novel insights into the *in vivo* kinetic host reactions against WNV infection and the pathophysiologic processes involved, according to clinical symptoms. This work offers useful clues for anti-viral research and further evaluation of early biomarkers for the diagnosis and prevention of severe neurological disease caused by WNV.

## Introduction

West Nile virus (WNV) is a small, enveloped, positive-stranded RNA virus belonging to the *Flaviviridae* family (genus *Flavivirus*). This arbovirus is widespread, occurring on all continents with the exception of Antarctica [1]. Since its isolation from a febrile woman in Uganda in 1937 [2], WNV has been responsible of high morbidity and mortality in infected birds, horses and humans [3]. WNV is maintained in nature in enzootic cycles involving mainly ornithophilic mosquitoes, predominantly *Culex* species, and avian hosts. Transmission to other vertebrates, such as horses or humans, occurs incidentally [4]. Therefore, horses or humans are considered incidental or “dead-end” hosts due to the insufficient blood viremia to infect a naïve feeding mosquito. Although human WNV infections are asymptomatic in more than 80% of cases, 1% of persons with clinical illness could develop neurologic symptoms such as meningitis, encephalitis and acute flaccid paralysis, with low mortality rates [5]. Severe disease may be life-threatening to susceptible individuals such as the very young, the elderly and immunocompromised patients [6].

The epidemiology of WNV changed in the 1990s and is now characterized by the increasing incidence of neuroinvasive symptoms in humans in the Mediterranean basin (*i.e.,* Algeria, Tunisia, Italy, Romania, Israel, France, Portugal, Spain and Hungary) and Russia [7], [8], [9], [10], [11], [12]. Moreover, the emergence of WNV in the summer of 1999 in the U.S. was responsible for the largest arboviral epidemic of human encephalitis in history, and it continues to be the most frequent cause of epidemic meningoencephalitis in North America [13]. Since then, WNV has spread throughout the American continent and has recently been reported in Mexico, South America, and the Caribbean [14]. WNV is now considered endemic in Africa, Asia, Australia, the Middle East, Europe and the United States [15]. In 2010, WNV emerged in Greece, resulting in 262 confirmed cases with 81 patients presenting with neurological manifestations and mortality rates of 9.9% [16]. Two main phylogenetic lineages of WNV have been described [17], with a higher incidence of neuroinvasive disease associated with subtypes of WNV lineage I. Lineage I strains have been more frequently detected in the recent European outbreaks [18].

The changing epidemiology, the constant risk of (re-)emergence of more virulent strains, and the lack of effective antiviral therapy or vaccines, makes understanding the pathogenesis of severe disease a priority [5]. Recent technological advances in genomics and proteomics have greatly improved our knowledge of the pathophysiological processes following virus infections [19], [20]. For example, DNA microarrays have been utilized to dissect the transcriptomic profiles in cultured cells infected with WNV [21] and to compare the neurovirulence of different WNV strains [22]. The authors showed that several genes involved in antiviral responses were up-regulated following WNV infection. Several genes involved in interferon-stimulated genes (ISGs), development of the immune response and cell apoptosis were differentially expressed. These molecular changes could represent different functions, some contributing to neurovirulence and others participating in the response to infection. Similarly, a global transcriptional analysis of human glioblastoma cells infected with WNV reported differential expression of 173 host genes, among which a subset participated in the regulation of diverse physiologic processes, such as immunity, apoptosis, the ubiquitin cycle and the regulation of transcription [23]. Recently, gene expression profiles in the central nervous system from horses infected with WNV were compared [24]. Significant changes were detected according to WNV exposure and/or vaccination, particularly in neurological, immunological and apoptotic pathways.

At the protein level, *in vitro* experiments carried out on Vero and neuronal cell cultures showed profound host modifications of the proteome following WNV infection [25], [26]. It was shown that a large majority of proteins were up-regulated, including proteins belonging to the apoptotic pathway, cell cycle regulation or maintenance of the cytoskeleton network. Although the precise mechanisms of these proteome alterations are still unclear, these changes could be attributed to two complementary phenomena: a viral manipulation of host proteins to successfully complete viral replication or a response from the host to counteract viral infection. However, analyses of the transcriptomic and proteomic profiles in the brain of susceptible animals after infection with WNV, considering clinical onset evolution, have not yet been described. Elucidating the protein interaction networks and the use of pathway-based analysis may provide an effective approach to investigate the molecular mechanisms of WNV neuroinvasive disease.

To gain insights into the pathophysiological processes in severe WNV infection, a kinetic analysis of protein expression profiles in the brain of WNV-infected mice was conducted using samples prior to and after the onset of clinical symptoms. To this end, 2D-DIGE and gel-free iTRAQ labeling approaches were combined, followed by protein identification by mass spectrometry. Using these quantitative proteomic approaches, a set of altered proteins was identified. The dataset was analyzed using ingenuity pathway analysis (IPA), which enabled the identification of functional signaling networks in samples collected during early and late infection. The results were subsequently translated into biological processes that may be involved in the pathogenesis of neuroinvasive disease caused by WNV infection.

## Materials and Methods

### Ethics Statement

All animal experiments described in this paper have been conducted according to Dutch guidelines for animal experimentation and approved by the Animal Welfare Committee of the Erasmus Medical Centre, Rotterdam, Netherlands. All efforts were made to minimize animal suffering.

### Reagents

N-hydroxy succinimide ester Cy2, Cy3 and Cy5, urea, glycerol, mineral oil, immobiline DryStrip gel (18 cm, pH = 3–10, pH = 4–7 and pH = 6–11) and IPG buffer solutions (pH = 3–10, 4–7 and 6–11) were purchased from GE Healthcare (Piscataway, NJ). Acrylamide, DTT, Tris, glycine and SDS were purchased from Bio-Rad (Hercules, CA, USA). Dimethyl formamide (DMF), CHAPS, L-lysine, ammonium persulfate, iodoacetamide, agarose, bromophenol blue and TFA were purchased from Aldrich (Poole, Dorset, UK). Thiourea, TEMED, acetone, acetonitrile (ACN) and ethanol were purchased from Fluka (Buchs, Switzerland). Trypsin (sequencing grade) was purchased from Promega (Madison, WI). All buffers were prepared with Milli-Q water (Millipore, Belford, MA, USA). Imperial™ Protein Stain solution was purchased from Thermo Scientific (Rockford, IL, USA).

### Cells and Virus

Vero E6 cells were grown in DMEM (Lonza Benelux BV, Breda, Netherlands) supplemented with antibiotics, 10% heat inactivated fetal calf serum (FCS), sodium bicarbonate and 10 mM HEPES buffer (all from Lonza). The prototype NY99 WNV strain (accession AF196835.2, obtained from the Health Protection Agency, Porton Down, UK; P5 on Vero E6 cells) was used for the infection of mice. The 50% tissue culture infectious dose (TCID50) was determined on VeroE6 cells using the Spearman & Kärber method based on the presence of cytopathic effects five days post inoculation [27], [28].

### Mouse Infection

Twenty nine-day old female C57/BL6 mice (Harlan Laboratories B.V., Venray, Netherlands) were inoculated intraperitoneally (i.p.) with 10^5^ TCID_50_ WNV-NY99. Five animals were euthanized by cervical dislocation under isoflurane anesthesia on days 3, 4, 5 and 6 post-inoculation, and brains were collected for further processing to determine virus titers in the brain. The results of this first kinetics mouse experiment were used to determine the early and late time-point of virus infection in the brain. Following this experiment, 12 nine-day old female C57/BL6 mice were inoculated i.p. with 10^5^ TCID_50_ WNV-NY99. Six animals infected with WNV were euthanized by cervical dislocation under isoflurane anesthesia on days 3 (early; WNV-E1 to E6) and 5 (late; WNV-L1 to L6) post-infection. Six animals were inoculated with DMEM (mock group; C1 to C6) and euthanized on day 2 to eliminate the influence of LPS production incurred by damage as a result of the injection. Brains were collected following euthanization, and half of the brain (left hemisphere; no cerebellum) was washed gently once with ice-cold PBS and frozen at −80°C until further use (see *Protein sample preparation* below). Mice were maintained in isolator cages throughout the infection experiment, had a 12-hour day-night cycle and were fed *ad libitum*. Animal experiments were approved by the Animal Ethics Committee of Erasmus Medical Center.

### Quantitation of Virus in the Brain

To quantify viral burden in the brain from the first kinetics mouse experiment, a half brain was weighed and homogenized using a metal bead in 1 mL DMEM containing antibiotics (100 U penicillin, 100 µg/mL streptomycin) using a tissue homogenizer. Brains of infected animals euthanized at the early and late time-point were removed and stored in Ambion® RNA-later (Invitrogen, Bleiswijk, Netherlands). RNA was isolated from the brain tissues using Trizol reagent (Invitrogen) and the RNeasy Mini kit (Qiagen). WNV RNA copy numbers in the brain were determined using positive-sense strand-specific qRT-PCR, as described by Lanford et al. [29], with the Taqman® EZ RT-PCR kit (Applied Biosystems, Bleiswijk, Netherlands) and primers and probe located on the 3′UTR of WNV. RNA copy numbers were quantified using a standard curve of *in vitro* transcribed RNA of known quantities. Run-off transcripts were generated from a plasmid containing the sequence of the 3′ UTR of WNV-NY99. Briefly, ‘tagged primers’ were generated by adding a 32-mer-long sequence of the Grapevine virus A as a tag at the 5′-end of the respective primers. Specific detection of the WNV-positive RNA strand was performed after cDNA synthesis using a tagged reverse primer, complimentary to the positive-sense strand and subsequently, the positive strand was amplified using the tagged sequence as the reverse primer and a WNV-specific unmodified forward primer. This procedure has been reported to prevent the amplification of cDNA products that are made by the false priming of either the positive or negative RNA strand as well as the amplification of cDNA acquired as a result of self-priming [29]. All RT reactions contained 30 pmol primer and were carried out for 2 min at 50°C and 30 min at 60°C using the r*Tth* RT enzyme according to the instructions provided by the Taqman® EZ RT-PCR kit (Applied Biosystems).

### Immunohistology

Sagittal brain 4-µm thick paraffin sections were processed for streptavidin-biotin-peroxidase immunohistochemistry of virus nonstructural protein. Sections were deparaffinized in xylene, rehydrated in descending concentrations of ethanol and incubated for 10 min in 3% H_2_O_2_ diluted in PBS to block endogenous peroxidase activity. Antigen exposure was performed by incubation for 15 min at 121°C in citrate buffer (0.01 M, pH 6.0). Sections were incubated overnight at 4°C with a primary goat anti-WNV nonstructural protein 3 (NS3) antibody (1∶100; R&D Systems, Abingdon, UK) and were detected using a secondary rabbit anti-goat IgG-peroxidase antibody (Dako, Eindhoven, The Netherlands). Sections were counterstained with Mayer’s hematoxylin and mounted with Kaiser’s glycerin-gelatin and analyzed using a light microscope.

### Protein Sample Preparation

For protein preparation, each brain sample was lysed with 1 ml of lysis buffer containing 2% SDS, 125 mM Tris-HCl pH = 6.8, 10% glycerol and 5% mercaptoethanol, and homogenised by mechanical disruption using metal beads and the Tissue Lyser apparatus (QIAGEN). The resulting homogenates were centrifuged for 15 min at 16 000×g at 4°C and the supernatant was collected and stored at −80°C. The protein concentration of each sample was determined by the Lowry method (DC Protein assay Kit, Bio-Rad) according to the manufacturer’s instructions. Protein concentration of each mouse brain sample homogenate ranged from 7.3 to 14.3 mg/ml.

### CyDye Labeling

Samples were subjected to 2-D clean-up kit (GE healthcare), concentrated by precipitation with acetone (Sigma, St. Louis, MI), and the protein pellet was resuspended at a protein concentration of 2.5 µg/µL in standard cell lysis buffer (UTC buffer) containing 8 M urea, 2 M thiourea, 4% (w/v) CHAPS and 30 mM Tris, adjusted to pH 8.5 as previously described [30]. Sample quality and protein amount was checked by loading 10 µg of each sample onto a 10% SDS-PAGE precast gel (Bio-Rad) stained with Imperial™ Protein Stain solution (Fisher Scientific) (data not shown). Proteins in each sample were minimally labeled with CyDye according to the manufacturer’s recommended protocols and as previously described [25]. Briefly, 50 µg of each protein sample were labeled with 400 pmol of either Cy3 or Cy5, freshly dissolved in anhydrous dimethylformamide, and incubated on ice for 30 min in the dark. The reaction was quenched with 1 µL of 10 mM free lysine by incubation 10 min on ice in the dark. An internal standard pool was generated for each study by combining an equal amount (25 µg) of each sample included in the study and was labeled with Cy-2. Cy3-, Cy5- and Cy2-labeled samples were then pooled (Tables S1 and S2), and an equal volume of UTC buffer containing 10 mM DTT and 1% (v/v) immobilized pH gradient (IPG) buffer corresponding to the IPG strips used, was added.

### Two-dimensional Electrophoresis

For the first dimension, labelled-samples were separated by isoelectric focusing (IEF) with precast 18-cm IPG strips with different pH gradient ranges (3–10 L, 4–7 or 6–11), rehydrated for 6 hours with DeStreak rehydration solution containing 1% (v/v) IPG buffer (pH 3–10 L, 4–7 or 6–11). The samples were applied at the acidic end of the IPG strips using a cup-loading technique. IEF was carried out at 20°C for a total of 55 kVh on an Ettan IPGphor 3 electrophoresis unit (GE Healthcare). Prior to separation in the second dimension, the IPG strips were reduced in Equilibration buffer (50 mM Tris-HCl, pH 8.6 buffer, 6 M urea, 2% SDS and 30% glycerol) supplemented with 1% (w/v) DTT for 10 min and then alkylated in Equilibration buffer containing 2.5% (w/v) iodoacetamide for 10 min. Equilibrated IPG strips were then deposed onto 10% SDS-PAGE gels using Ettan DALT six system (GE Healthcare UK). Strips were overlaid with 0.5% low-melting point agarose in 1× running buffer containing bromophenol blue and electrophoresis was run overnight at 20°C, 1.5 W/gel, until the dye reached the bottom of the gel.

### Image Analysis

After electrophoresis, the gels with Cydye-labeled proteins were scanned three times with a Typhoon™ Trio Image scanner (GE Healthcare UK) each time at different excitation wavelengths (Cy3, 580 BP 30/green (532 nm); Cy5, 670 BP 30/red (633 nm); Cy2, 520 BP 40/blue (488)). Pre-scans were performed to adjust the photomultiplier tube (PMT) voltage to obtain images with a maximum intensity of 60 000 to 80 000 U. Images were cropped with ImageQuant™ software (GE Healthcare UK) and further analyzed using the software package Progenesis SameSpot v2 software (Nonlinear Dynamics, Newcastle upon Tyne, UK). The gel images were aligned by automated calculation of alignment vectors after assigning landmark vectors. Background subtraction and spot intensity normalization were automatically performed by Progenesis SameSpots. Protein spots which presented a significant abundance variation between the 3 experimental groups (|ratio| ≥1.3, ANOVA *p*≤0.05) were marked and submitted to mass spectrometry for identification.

### In-gel Digestion

Based on the Progenesis SameSpot analysis, protein spots of interest from gels stained with Imperial™ Protein Stain solution were excised and digested using a Shimadzu Xcise automated gel processing platform (Shimadzu Biotech, Kyoto, Japan) as described previously [31] and stored at −20°C until their analysis by mass spectrometry (MS).

### Mass Spectrometry Analysis of Peptide Mixture from Gel Elution and MS Data Analysis

The samples were subjected to nanoscale capillary liquid chromatography-tandem mass spectrometry (nano LC-MS/MS) analysis with a QTOF apparatus (Q-TOF Ultima, Waters, MA) as previously described [25]. The peak lists generated in the micromass pkl format, were then fed into a local search engine Mascot Daemon v2.2.2 (Matrix Science, London, UK) against a mixed *Mus musculus* and West-Nile virus homemade protein database (SwissProt). Search parameters were set in order to allow one missed tryptic cleavage site, the carbamidomethylation of cysteine, and the possible oxidation of methionine; precursor and product ion mass error tolerance was <0.2 Da. All identified proteins have a Mascot score greater than 34 (Mixed: *Mus musculus* and West-Nile virus, 16414 sequences June 26^th^, 2012), corresponding to a statistically significant (*p*<0.05) confident identification. Moreover, among the positive matches, only protein identifications based on at least two different non-overlapping peptide sequences with a mass tolerance <0.05 Da were accepted. For single peptide-based identification, in addition to Mascot score significance, only peptide sequence with at least six consecutive amino acids detected on MS spectra were considered. These additional validation criteria struck a balance that limited the number of false positive matches without missing real proteins of interest.

### iTRAQ Labeling

For iTRAQ labeling, sample pool of each experimental group was generated by mixing an equal amount of each sample per group (control, pool-C; early, pool-E; and late, pool-L). Six mice per group were pooled. Each pool was divided either in four (control, pool-C1-C4) or 2 (pool-E1-E2 and pool-L1-L2) replicates containing 100 µg protein. Proteins were precipitated with cold acetone for 2 h at –20°C, centrifuged for 15 min at 16 000× *g*, dissolved in 20 µL of Dissolution buffer, denatured, reduced, alkylated and digested with 10 µg of trypsin overnight at 37°C, following manufacturer’s protocol (iTRAQ® Reagent Multiplex Buffer kit, Applied Biosystems, Foster City, CA, USA) and as previously described [32]. The resulting peptides were labeled with iTRAQ reagents (iTRAQ® Reagent-8Plex multiplex kit, Applied Biosystems) according to manufacturer’s instructions. Peptides from the 4 mock samples (pool-C1-C4) were labeled with 113 to 116 iTRAQ reagents, peptides from the 2 early WNV-infected samples (pool-E1-E2) were labeled with 117 and 118 iTRAQ reagents and peptides from the 2 late WNV-infected samples (pool-L1-L2) were labeled with 119 and 121 iTRAQ reagents (Table S3) at room temperature for 2 h and stored at −20°C. Before combining the samples, a pre-mix containing an aliquot of each sample, cleaned-up using a ZipTip®, was analyzed by MS/MS to check for peptide labeling efficiency with iTRAQ reagents and homogeneity of labeling between each sample. The content of each iTRAQ reagent-labeled sample was then pooled into one tube according to this previous test. The mixture was then cleaned using an exchange chromatography (SCX/ICAT cation exchange cartridge, AB Sciex, Foster City, USA) and reverse-phase chromatography C18 cartridge (C18 SpinTips, Protea bio, Nîmes, France) prior to separation using an off-gel system (Agilent 3100 OFFGEL fractionator, Agilent Technologies).

### Off-gel Separation

The resulting peptides were dried and separated into 12 fractions in-solution with an Agilent 3100 OFFGEL fractionator (Agilent Technologies). Peptides separation was based on their isoelectric point on a 13-cm IPG strips pH 3–10 using IPG buffer, pH 3–10 (Agilent Technologies). The IPG strips and paper wicks were rehydrated with 40 µl of 2.44% glycerol (v/v), 1% IPG buffer for 15 min. While the strips were rehydrating, the sample was solubilized in 1.8 ml of the same rehydration buffer. After complete rehydration, 150 µl of sample was added to each well, the wells were sealed, and mineral oil was added to each end of the strip. The strips were focused until 20 kV h was reached with a max voltage of 8000 V, 50 µA, 200 mW, and a hold setting of 500 V. After 24 h of running time the paper wicks were changed with new wicks wetted with water. The runs took approximately 35–40 h.

### Mass Spectrometry Analysis of Peptide Fractions from Off-gel Separation

For nanoLC mass spectrometry measurements, approximately 5 µg of peptide sample was injected onto a nanoliquid chromatography system (UltiMate® 3000 Rapid Separation LC (RSLC) systems, Dionex, Sunnyvale, CA). After pre-concentration and washing of the sample on a Dionex Acclaim PepMap 100 C18 column (2 cm × 100 µm internal diameter (id) 100 A, 5 µm particle size), peptides were separated on a Dionex Acclaim PepMap RSLC C18 column (15 cm × 75 µm id, 100 A, 2 mm particle size) (Dionex, Amsterdam) using a linear 180 min gradient (4–40% acetonitrile/H20; 0.1% formic acid) at a flow rate of 300 nL/min. The separation of the peptides was monitored by a UV detector (absorption at 214 nm). The nanoLC was coupled to a nanospray source of a linear ion trap Orbitrap mass spectrometer (LTQ Velos Orbitrap, Thermo Electron, Bremen, Germany). The LTQ spray voltage was 1.4 kV and the capillary temperature was set at 275°C. All samples were measured in a data dependent acquisition mode. Each run was preceded by a blank MS run in order to monitor system background. The peptide masses were measured in a survey full scan (scan range 300–1700 m/z, with 30 K FWHM resolution at m/z = 400, target AGC value of 10^6^ ions and maximum injection time of 500 ms). In parallel to the high-resolution full scan in the Orbitrap, the data-dependent HCD scans of the 10 most intense precursor ions were fragmented and measured in the orbitrap analyser (normalized collision energy of 35%, activation time of 10 ms target AGC value of 10^4^, maximum injection time 100 ms, isolation window 2 Da and wideband activation enabled). Dynamic exclusion was implemented with a repeat count of 1 and exclusion duration of 30 sec.

### Data Analysis

Raw files generated from mass spectrometry analysis were combined and processed with Proteome Discoverer 1.3 (Thermo Fisher Scientific). This software was used for extraction of MGF files. Protein identification and quantification were carried out using ProteinPilot version 4.0 (Applied Biosytems). The search was performed against the mixed database containing 56478 sequences (*Mus musculus* (54080 sequences from Uniprot December 13^rd^, 2011)+West Nile virus (2242 sequences), and some classical contaminant proteins (156 sequences)). Data were processed as described previously [33].

### SDS-PAGE, Blotting, and Analysis Procedures

Immunoblotting with fluorescence-based methods was used to detect both the total protein expression profile and the specific immunoreactive proteins, as described previously [25]. The same protein samples used for proteomic analysis were minimally labeled with CyDye (*i.e.,* Cy3) as described above (see “CyDye Labeling” section). Labeled samples were separated by 10% or 4–20% SDS-PAGE in a Mini-PROTEAN Cell (Bio-Rad) according to the molecular weight of the targeted proteins. Gels were transferred to a nitrocellulose membrane (0.45 µm; GE Healthcare) using a semidry blotting system at 200 mA for 30 min (TE 77 PWR Semi-Dry Transfer Unit, GE Healthcare) [34]. Blots were saturated with 5% nonfat dried milk in PBS containing 0.05% (v/v) Tween 20 (PBS-T-milk) for 1 h. Western blot (WB) analyses were carried out with rabbit mono- or polyclonal antibodies directed against microtubule-associated protein 2 (1∶400, MAP2, no. sc-20172, Santa Cruz Biotechnology, Inc., Santa Cruz, CA), microtubule-associated protein 1B (1∶400, MAP1B, no. sc-25729, Santa Cruz), clathrin heavy chain (1∶500, CLTC, no. 2410, Cell Signaling Technology, Danvers, MA), dynamin 1 (1∶1000, DNM1, no. sc-6402, Santa Cruz), vimentin (1∶500, VIM, no. 3932, Cell Signaling), E3 ubiquitin-protein ligase HUWE1 (1∶200, UREB1, no. sc-134821, Santa Cruz), signal transducer and activator of transcription 1 (1∶1000, STAT1, no. 9172, Cell Signaling), phosphotyrosine701-STAT1 (1∶500, phospho-STAT1 (Tyr701), no. 9167, Cell Signaling), STAT2 (1∶100, STAT2, no. sc-22816, Santa Cruz), peroxiredoxin 6 (1∶100, PRDX6, no. sc-134478, Santa Cruz), glial fibrillary acidic protein (1∶1000, GFAP, no. Z0334, DAKO, Glostrup, Denmark), or with a goat polyclonal antibody directed against calpain 9 (1∶400, CAPN9, no. sc-66508, Santa Cruz), diluted in PBS-T-milk and incubated overnight at 4°C. After three washes in PBS-T, primary rabbit antibodies were revealed with ECL Plex goat anti-rabbit IgG Cy5-conjugated secondary antibody (1∶1000, GE Healthcare), and rabbit anti-goat IgG fluorescein isothiocyanate (FITC) conjugate (1∶400, Southern Biotech, Birmingham, AL) was used for the detection of anti-CAPN9 goat antibody diluted in PBS-T-milk. All manipulations were protected from light. As a positive control for the p-701-STAT1 antibody response, a lysate of murine bone marrow-derived macrophages stimulated for two hours with IFN-γ (PeproTech; 20 ng/ml) was used. The gels electrophoresis and immunoblots were scanned using a Typhoon Trio image scanner as mentioned above (see “Image Analysis” section). Immunoreactive bands were analyzed using TotalLab Quant v12.2 software (Nonlinear Dynamics). To evaluate the expression level of the different proteins, immunoreactive band intensities were normalized to the intensities of a global protein pattern labeled with Cy3 as described previously [25]. Band intensities were also corrected for the adjacent background. Differences in the relative abundance of each protein between two independent groups were determined using Student’s *t*-test. All differences were considered significant at *p*<0.05 and statistical analysis was performed using GraphPad Prism v5.01 statistical software (GraphPad Software Inc., La Jolla, CA). Standard molecular weight markers were loaded in each gel (Bio-Rad).

### Ingenuity Pathway Analysis

A dataset containing differentially regulated proteins obtained from the 2D-DIGE and iTRAQ analysis and their corresponding expression values (fold-change and *p*-values) were uploaded into the IPA software, Inc. (http://www.ingenuity.com). In this analysis, the evolution of protein expression was taken into account according to the onset of clinical symptoms. Proteins whose expression was significantly differentially regulated (fold-change ≥1.3, *p*-value ≤ 0.05) were selected for the analysis. The IPA program uses a knowledgebase (IPA KB) derived from the scientific literature to connect genes or proteins based on their relationships and functions. Ingenuity Pathway Analysis generates biological networks, canonical pathways and functions that are relevant to the uploaded dataset. A right-tailed Fisher’s exact test is used for calculating *p*-values to determine if the probability that the association between the proteins in the dataset and the functional and canonical pathway can be explained by chance alone. The scores are derived from a *p*-value (score = −log (*p*-value)) and indicate the likelihood that the proteins of interest (*i.e.,* the identified proteins within a network)) are clustered together. Thus, these proteins and their association with the IPA KB were used to generate networks and to perform functional canonical pathway analyses.

## Results

### Virus Kinetics Study

**Figure 1:**
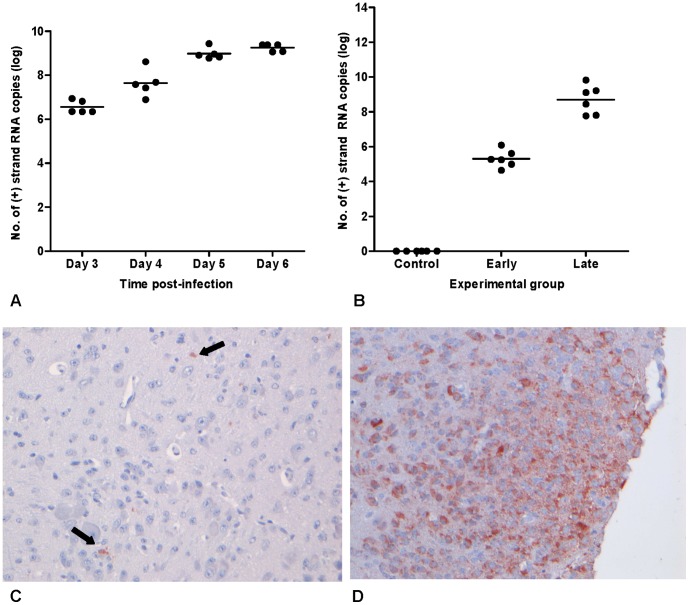
Detection of WNV in brains of mice infected with WNV-NY99. (A) The amount of WNV positive-sense RNA copies present in the brains of mice between days 3 and 6 post-infection with WNV-NY99. Mice were euthanized on day 3, 4, 5 and 6 post-infection and brains were collected and homogenized in DMEM for RNA isolation. (B) The amount of positive-sense RNA copies present in the brains of control mice and mice inoculated on days 3 and 5 post-infection. Brains were collected in RNA-later for RNA isolation using Trizol. Histopathology of the brains of 9-day old C57BL/6 mice infected with WNV-NY99. (C) Infected neurons in the brain of a mouse euthanized on day 3 p.i. (objective 20x). (D) Infected neurons in the cortex of a mouse euthanized on day 5 p.i. (objective 20x). Pictures are representative of the number of infected cells found in the brain for the majority of mice.

To determine the time-point at which the virus is first detected in the brain after peripheral inoculation (*i.e.,* early time-point, WNV-E) and the time-point of advanced disease (*i.e.,* late time-point, WNV-L) for the collection of brain samples, a kinetics experiment was carried out in which mice were infected and subsequently euthanized on day 3, 4, 5 and 6. On day 3, mice did not yet display clinical symptoms, but on day 4 after infection, mice first started to show signs of illness such as lethargy, decreased mobility and balancing problems, and by day 6 they displayed signs of immobility and paralysis. Virus titers in the brains of these mice were determined by qRT-PCR and infection was confirmed at all four time-points (Figure 1A). On day 3, approximately 6.5 log_10_ of positive strand RNA copies were present in the brain, which steadily increased until day 6 to approximately 9 log_10_ RNA copies. Based on these results, we determined the early time-point of infection to be on day 3 and the late time-point to be on day 5. All mice euthanized at the early and late time-point were positive for virus in the brain with mean virus titers of approximately 5.3 log_10_ and 8.7 log_10_ positive strand RNA copies, respectively (Figure 1B). All control mice were negative for virus in the brain. Infection of the brain was also confirmed using immunohistochemical staining with an anti-WNV-NS3 polyclonal antibody. Viral antigen was not detected in brain samples collected on day 1 or 2 after infection. In contrast, low amount of virus was demonstrated in the brains of mice at the early time-point (Figure 1C), whereas more extensive staining was found at the late time-point (Figure 1D), in line with the PCR results. It cannot be excluded that the viral RNA detected in the brain contained spill-over virus from the blood. However, the aim of the RT-PCR was to confirm that presence of viral antigen was associated with presence of viral RNA.

### Detection of Differentially Expressed Proteins Following WNV Infection by 2D-DIGE Analysis

To determine the proteins that were differentially regulated following WNV infection, half whole brain hemispheres from WNV-infected mice were sampled at early (E) and late (L) time-points, and protein extracts were analyzed by 2D-DIGE (Tables S1 and S2). Brains from non-infected mice (mock) were used as controls. Six mice per group were tested.

**Figure 2:**
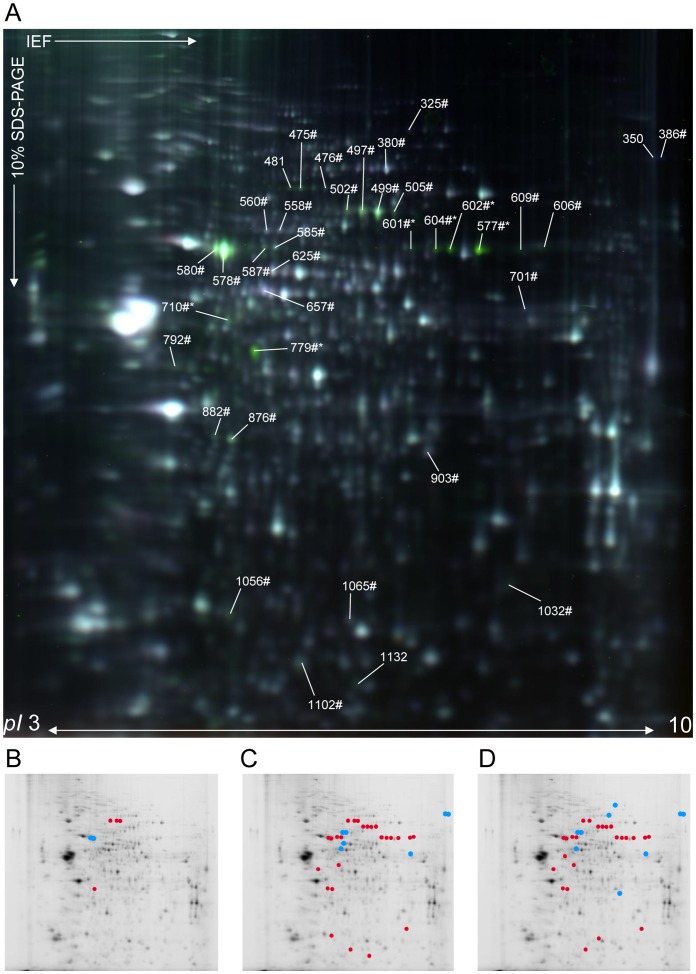
2D-DIGE analysis (pH 3–10) of mock-, early and late WNV-infected brain samples. (A) Representative data from a 2D-DIGE experiment using a 10% SDS-polyacrylamide gel with the pH range from 3 to 10 are shown. Proteins from mock- and late WNV-infected brain samples were labeled with Cy5 and Cy3 cyanine dyes, respectively. As determined by Progenesis SameSpot software, protein spots that were differentially regulated between the three experimental conditions (|ratio| ≥1.3 and *p*≤0.05), were submitted to mass spectrometry for identification. The numbers annotated on the gel correspond to master gel numbers of differentially regulated protein spots. Spots identified as *Mus musculus* (numbers#) and WNV (numbers*) proteins are listed in Table 1. Spots identified as a mixture of both species are indicated by numbers#*. Spots that were differentially modified between WNV-early and mock (B), WNV-late and WNV-early (C) and WNV-late and mock (D) infected samples are represented by red (up-regulated) or blue (down-regulated) dots.

Using the Progenesis SameSpot v.2 software, the abundance of 37 protein spots was found to be significantly changed (ANOVA, *p*≤0.05) between the three groups (mock, WNV-E and WNV-L) with a fold-change ≥30% (|FC| ≥1.3) in the pH range 3–10 (Figure 2A). The major portion of the protein spots were significantly altered at the late time-point compared to both the mock condition (85%, n = 32; 25 up- and 7 down-regulated, Figure 2D) and the early-groups (81%, n = 34; 25 up- and 9 down-regulated, Figure 2C). The comparison of protein profiles between the early- and mock-groups allowed the detection of six protein spots representing significant changes (4 up- and 2 down-regulated, Figure 2B). Because the determination of host proteome alterations prior to the appearance of clinical symptoms after WNV infection was one of the main aims of the present study, 2D-DIGE analyses were performed on early-infected samples compared to mock-infected samples using narrower pH range IPG strips (pH 4–7 and 6–11) to enhance and increase protein resolution. Using pH 4–7 IPG strips for the IEF, 19 protein spots were found to be significantly altered between early WNV- and mock-infected samples (fold-change ≥1.3, *p*≤0.05) (14 up- and 5 down-regulated, Figure S1). Although no protein spots were found to be significantly differentially regulated using pH 6–11 IPG strips (data not shown), the use of a narrower pH range allowed a three-fold increase in the number of differentially regulated protein spots at the early time-point compared to the mock group. Overall, a total of 56 distinct protein spots were found to be differentially regulated in the brain following WNV infection.

### Identification of Altered Protein Abundance Following WNV Infection from 2D-DIGE Analysis

The 56 protein spots of interest were excised from gels, subjected to in-gel digestion, and then analyzed by tandem MS for identification. The resulting fragment ion spectra were searched against *Mus musculus* and West Nile virus protein databases (SwissProt). Among the 37 protein spots selected from the 3–10 pH range analysis, 34 (91.9%) were successfully identified with a high degree of confidence, corresponding to 25 distinct proteins according to their accession number (Table 1). These proteins were grouped into functional categories according to gene ontology (GO). Multiple proteins were present in some of these spots; one spot contained a mixture of viral and human proteins (*e.g.,* spot ID: 710), and some proteins were present in multiple spots (*e.g.,* Dynamin-1 was detected in spot IDs: 475 and 476 in the pH range 3–10, Figure 2A and Table 1). As expected, the most important average fold-change corresponded to WNV proteins (mean ± SD: 3.8±1.8, Table 1). Interestingly, despite a detection of viral RNA in the brain of all animals prior to and after clinical onset (Figure 1), viral proteins were identified only at the late time-point, indicating that at the early time-point, viral proteins are below the detection limit of this method. Three protein spots could not be identified, most likely due to an insufficient amount of protein and/or low MS spectra quality.

**Table 1:** Proteins identified from the differential 2D DIGE analysis (pH 3–10) after WNV infection.

Accession number (SwissProt)	Protein name	Molecular weight (kDa)	*pI*	Spot ID	Number of MS/MS peptide sequences	Sequence coverage (%)	Mascot score	Average volume ratio			Anova (*p-value*)
								WNV-E vs. mock	WNV-L vs. mock	WNV-L vs. WNV-E	
**Viral proteins**											
POLG_KUNJM	Genome polyprotein [Kunjin virus]	384.717	8.70	577	11	3.1	324		5.5	6.0	2.7e-8
				601	8	2.7	333		1.4	1.3	5.4e-5
				602	6	2.0	200		2.9	3.0	6.9e-6
				604	6	2.0	231		3.4	3.2	1.6e-6
POLG_WNV	Genome polyprotein [West Nile virus]	383.401	8.63	710	1	0.3	77			1.4	0.003
				779	1	0.3	98		6.4	5.4	4.3e-11
**Host proteins**											
Metabolic/biosynthertic process											
ACLY_MOUSE	ATP-citrate synthase [Mus musculus]	120.564	7.13	325	9	10.0	231			−1.4	0.005
BACH_MOUSE	Cytosolic acyl coenzyme A thioester hydrolase [Mus musculus]	42.966	8.90	903	3	8.7	70			−1.3	8.7e-7
CAH2_MOUSE	Carbonic anhydrase 2 [Mus musculus]	29.129	6.49	1065	1	3.5	35			1.3	0.003
CATA_MOUSE	Catalase [Mus musculus]	60.403	7.72	701	8	16.9	205		−1.3	−1.3	1.5e-6
GPDM_MOUSE	Glycerol-3-phosphate dehydrogenase, mitochondrial [Mus musculus]	81.416	6.17	1032	2	4.5	100		1.5	1.4	0.004
SYG_MOUSE	Glycyl-tRNA synthetase [Mus musculus]	82.624	6.24	560	5	6.4	105		−1.3	−1.3	2.1e-5
Host response/protein folding											
ALBU_MOUSE	Serum albumin [Mus musculus]	70.700	5.75	578	22	35.0	1468	−1.4	1.8	2.4	2.7e-7
				580	14	25.8	1535	−1.3	1.6	2.1	5.5e-7
				585	12	20.2	190		1.4	1.5	7.0e-6
				587	1	2.1	69		1.6	1.8	5.1e-7
				606	2	3.0	38		1.4	1.4	2.0e-6
				609	1	2.5	116		2.0	2.0	2.8e-7
HS12A_MOUSE	Heat shock 70 kDa protein 12A [Mus musculus]	75.167	6.32	558	4	7.6	103		−1.4	−1.4	4.1e-5
HSP7C_MOUSE	Heat shock cognate 71 kDa protein [Mus musculus]	71.055	5.37	578	10	19.0	231	−1.4	1.8	2.4	2.7e-7
				580	3	6.8	120	−1.3	1.6	2.1	5.5e-7
				585	11	21.1	273		1.4	1.5	7.0e-6
				587	1	2.3	40		1.6	1.8	5.1e-7
				609	1	1.9	76		2.0	2.0	2.8e-7
PRDX6_MOUSE	Peroxiredoxin-6 [Mus musculus]	24.969	5.71	1102	6	30.4	106		1.3	1.3	0.001
TCPA_MOUSE	T-complex protein 1 subunit alpha [Mus musculus]	60.867	5.82	657	4	8.1	187		−1.3	−1.3	0.024
TCPG_MOUSE	T-complex protein 1 subunit gamma [Mus musculus]	61.162	6.28	476	6	20.2	164	1.3	1.6		6.8e-5
Transcription/translation regulation											
EF2_MOUSE	Elongation factor 2 [Mus musculus]	96.222	6.41	380	21	25.3	609			−1.3	0.006
FUBP2_MOUSE	Far upstream element-binding protein 2 [Mus musculus]	77.184	6.90	505	9	15.0	191		1.4	1.4	2.2e-5
SFPQ_MOUSE	Splicing factor, proline- and glutamine-rich [Mus musculus]	75.508	9.45	386	3	3.4	46		−2.0	−1.6	1.2e-4
Cytoskeleton maintenanvce											
ACTB_MOUSE	Actin, cytoplasmic 1 [Mus musculus]	42.052	5.29	876	3	7.7	48	1.3	1.8	1.4	2.9e-8
				882	8	25.9	241		1.6	1.4	2.1e-4
				1056	3	7.7	58		1.4		9.1e-4
DYN1_MOUSE	Dynamin-1 [Mus musculus]	98.140	7.61	475	9	13.0	170	1.3	1.6	1.3	3.6e-5
				476	5	15.6	163	1.3	1.6		6.8e-5
TBB5_MOUSE	Tubulin beta-5 chain [Mus musculus]	50.095	4.78	710	6	15.0	225			1.4	0.003
Nervous system developement											
DPYL2_MOUSE	Dihydropyrimidinase-related protein 2 [Mus musculus]	62.638	5.95	558	9	20.1	510		−1.4	−1.4	4.1e-5
				560	9	20.1	298		−1.3	−1.3	2.1e-5
				625	12	26.7	739		−1.3		2.0e-6
DPYL3_MOUSE	Dihydropyrimidinase-related protein 3 [Mus musculus]	62.296	6.04	657	13	29.1	989		−1.3	−1.3	0.024
GFAP_MOUSE	Glial fibrillary acidic protein [Mus musculus]	49.927	5.27	792	16	34.5	385		1.4	1.3	2.2e-5
Transport											
TRFE_MOUSE	Serotransferrin [Mus musculus]	78.841	6.94	497	12	17.9	467		1.5	1.9	1.5e-7
				499	16	25.4	616		1.6	2.0	5.4e-7
				502	12	22.8	428		1.5	1.6	4.5e-6
VATB2_MOUSE	V-type proton ATPase subunit B, brain isoform [Mus musculus]	56.857	5.57	710	2	4.1	70			1.4	0.003
Not identified											
	*n.i*			350					−1.4	−1.3	8.9e-5
	*n.i*			481				1.4	2.4	1.8	1.1e-9
	*n.i*			1132					1.3		0.002

The proteins were identified by mass spectrometry following in-gel trypsin digestion. The spot numbers correspond to the same numbers as indicated on Figure 2. The identities of the spots, their SwissProt accession numbers, and the theoretical molecular masses and *pI* values as well as the number of peptide sequences, the corresponding percent sequence coverage, and the Mascot score are listed for MS/MS analysis. Protein scores greater than 34 were considered as significant (*p<0.05*). Paired average volume ratio and *p* values (ANOVA) between the three experimental groups were defined using Progenesis Samespot software. Missing data correspond to paired average volume ratio not significantly changes. *n.i*., no identification.

**Figure 3:**
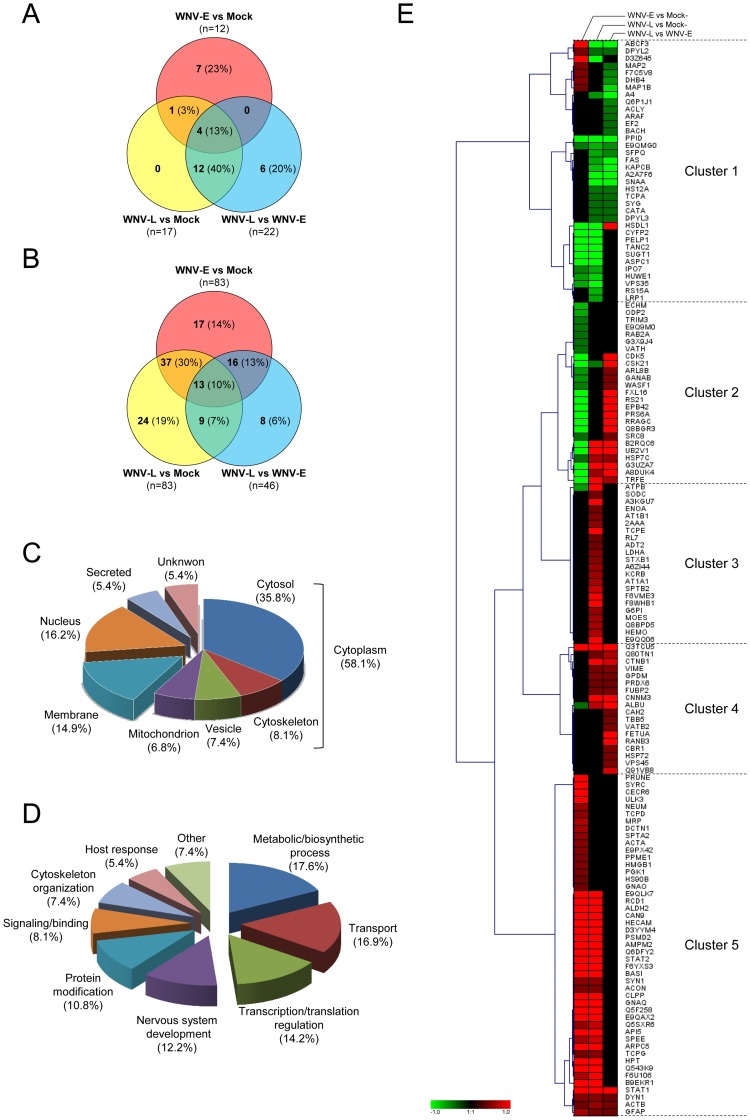
Classification of proteins significantly differentially regulated following WNV infection identified by 2D-DIGE and iTRAQ analysis. Venn diagram representing unique host proteins identified according to experimental group comparisons following WNV-infection by 2D-DIGE (A) and iTRAQ (B) analysis. The number of host proteins significantly differentially regulated between WNV-E and mock, WNV-L and mock or WNV-L and WNV-E are indicated. The two samples that were compared are indicated adjacent to each circle. The number and the percentage of proteins associated with each category are indicated in brackets. Classification of the significantly differentially regulated proteins according to their sub-cellular location (C) and their functional categorization (D) according to gene ontology. The percentages of proteins associated with each category are indicated in brackets. (E) Hierarchical clustering analysis was performed according to the (mean) ratios calculated between WNV-E and mock, WNV-L and mock or WNV-L and WNV-E, as indicated at the top of the graphic. Up- and down-regulated proteins are shown in red and green, respectively, and proteins with no change in expression level are indicated in black. The intensity of red or green color corresponds to the degree of regulation as indicated by the color strip at the bottom of the figure in arbitrary units. The graphical cluster was generated using the Genesis program [130].

Among the 19 protein spots selected from the 4–7 pH range analysis, 17 (89.5%) were identified corresponding to 10 distinct host proteins according to their SwissProt accession number (Table S4), and four of them were identified in more than one spot. As observed using the pH 3–10 analysis, at the early time-point, no viral proteins were identified. Among the five proteins identified at WNV-E with the pH 3–10 analysis, three proteins (serum albumin, dynamin 1 and T-complex protein 1 subunit gamma) were also characterized with the pH 4–7 analysis at the early time-point and were differentially regulated in the same way. Thus, a total of 12 distinct differentially regulated proteins were identified in the WNV-E samples before the appearance of clinical symptoms. Taking into account both pH ranges of the 2D-DIGE analyses, 30 unique significantly differentially regulated host proteins were identified. Of these proteins, some (ACTB, ALBU, DYN1 and HSP7C) were found to be commonly regulated between the different comparisons, and others appeared to be differentially regulated exclusively at the early or late time-points, as depicted in the Venn diagram (Figure 3A).

### Identification of Differentially Expressed Proteins Following WNV Infection using iTRAQ Labeling

To complete the overview of mouse brain proteome changes after WNV infection, an off-gel quantitative proteomic analysis was performed using iTRAQ reagents allowing the labeling and the comparison of 8 distinct samples. Then, the six brain samples from each experimental condition (*i.e.,* WNV-E, WNV-L and mock) were pooled and separated into four mock, two WNV-E and two WNV-L replicates, each labeled with a specific iTRAQ reagent (from 113 to 121 as indicated in Table S3). Labeled samples were mixed and separated by an off-gel system into 12 fractions prior to subjecting each fraction to tandem MS analysis. Data generated were analyzed with Protein Pilot software using the parameters described above. The application of a global False Discovery Rate (FDR) of 5% and the exclusion of classical contaminant proteins gave rise to a total of 1159 identified and quantified proteins that were included in the analysis.

Among them, a total of 124 distinct proteins were found to be modified (*p*≤0.05) between the three groups (mock, early and late) with a fold-change ≥30% (|FC| ≥1.3) (Table S5). Among them, 83 proteins were changed between WNV-E and mock-infected mice (47 up- and 36 down-regulated). Between the WNV-L and mock-infected mice, 83 proteins were found to be modified (62 up- and 21 down-regulated), and between the WNV-L and WNV-E time-points, 46 proteins was found to be modified (31 up- and 15 down-regulated). Among the 124 differentially regulated proteins, 13 were found commonly modified in the three comparisons, and 62 proteins showed modified expression in paired comparisons (Figure 3B). Additionally, 49 proteins were differentially regulated in only one comparison: 17 between the WNV-E and mock-infected mice, 24 between the WNV-L and mock-infected mice, and 8 between the WNV-L and WNV-E time-points.

### Combination of In-gel (2D-DIGE) and Off-gel (iTRAQ-labeling) Analyses

The two complementary quantitative proteomic approaches, 2D-DIGE and iTRAQ labeling, generated a total of 148 unique host proteins that were found to be differentially expressed in brain tissue samples after WNV infection at the early and/or late time-points. Six proteins (TRE, ALBU, DNM1, GFAP, TCPG and DPYSL2) were identified by both proteomic approaches and were differentially regulated in the same way. The cellular distribution analysis and functional annotation of these significantly differentially expressed proteins was performed (Figure 3C and 3D).

To obtain a better view of the expression profile of these differentially regulated proteins during the course of WNV infection, a cluster analysis was performed. Using a hierarchical clustering analysis, proteins that showed the same expression patterns during the WNV infection were grouped together (Figure 3E). Five clusters of expression pattern could be distinguished. Cluster 1 contains 36 proteins that are mainly repressed at the early and/or late time-points. Many of these proteins were involved in nervous system development, (*e.g.,* APP, MAP1B, MAP2, NCAM1 and DPYSL proteins), and transcription/translation regulation (*e.g.,* PELP1, EEF2 and RPS15A). Cluster 2 encompassed proteins whose expression was down-regulated at the early time-point and was subsequently unchanged or up-regulated compared to the earliest time-points. This cluster was composed of 25 proteins involved in transport and transcription/translation regulation. Clusters 3, 4 and 5 included 22, 18 and 47 proteins, respectively, that were up-regulated in at least one comparison. Proteins in cluster 3 were mainly associated with transport, proteins in cluster 4 were related to host response and proteins in cluster 5 were associated with protein modifications (*e.g.,* CCT proteins, HSP90 and UBE2M), metabolic process (*e.g.,* ALDH2, PGK1) and transcriptional regulation (*e.g.,* STAT1 and STAT2).

Interestingly, most of the 55 proteins that were found to be differentially regulated at the early and late time-points compared to mock were modified in the same direction (12 down- and 32 up-regulated proteins). Some of them showed an amplification of the differential regulation during the time-course of WNV-infection (early *vs.* mock and late *vs.* early), such as PPID and PCM1 (down-regulated) and TAPBP and STAT1 (up-regulated). The other 11 proteins were differentially regulated in opposing ways at the early and late time-points (Figure 3E, Tables 1 and 2, Table S5). Differentially regulated proteins and their respective biological functions and processes were further analyzed using bioinformatics to predict the possible effects of their altered abundance.

**Table 2:** Top three IPA-generated networks of differentially expressed molecules identified by DIGE and iTRAQ labeling between early and mock, late and mock, and late and early WNV-infected brain samples.

Top Functions	Score	Focus Molecules	Molecules in Network
***Early vs mock***			
Protein Synthesis, Cell Death, Cancer	49	23	*up-regulated*ACO2 (includes EG:11429), GFAP, HNRNPA2B1, HP, HSP90AB1, METAP2, PGK1, PRUNE, STAT1, STAT2, TAPBP*down-regulated*ALB, ARL8B, ATP5B, CAMK4, CSNK2A1, HBB, HSPA8, PPID, RNA RPS21, SUGT1, SUMO3, UBE2V1*not in the dataset*Creb, hemoglobin, Hsp70, Ifi47, ISGF3, IWS1, NLRP12, Nlrp4a, RNA polymerase II, Secretase gamma, Stat1-Stat2, UBE2F
Nervous System Development and Function, Organ Morphology, Cell Morphology	48	22	*up-regulated*ACTA2, ACTB, ACTG2, CCT3, CCT4, CLTC, DCTN1, DNM1, DPYSL2, GAP43, GNAQ, MAP2, MAP1B, MARCKSL1, NCAM1, SPTAN1, SYN1, TRRAP*down-regulated*CDK5, CYFIP2, EPB42, WASF1*not in the dataset*Actin, Alpha Actinin, Alpha tubulin, Beta Tubulin, Calmodulin, Clathrin, F Actin, G-Actin, Hsp90, PLC gamma, Profilin, Spectrin, Tubulin
Molecular Transport, Small Molecule Biochemistry, Cell Cycle	43	21	*up-regulated*API5, BSG (includes EG:12215), CAPN9, CECR6, GNAO1, Hmgb1 (includes EG:100041307), Hmgb1 (includes EG:25459), HSD17B4, OPCML, PNP, PSMD2, RQCD1, WDR72*down-regulated*CEP290, FBXL16, GANAB, HUWE1, PCM1, PELP1, PSMC3, TANC2*not in the dataset*ATP1B2, BCAS3, HTT, JKAMP, miR-30c-5p (and other miRNAs w/seed GUAAACA), miR-340-5p (miRNAs w/seed UAUAAAG), NDRG1, PAAF1, PPIC, PRNP, RB1, Serpina3g (includes others), TP53 (includes EG:22059), UBA5
***Late vs mock***			
Cellular Assembly and Organization, Nervous System Development and Function, Cell Death	48	22	*up-regulated*ACO2 (includes EG:11429), ACTB, CAMK2A, CCT3, CCT5, CLTC, DNM1, GNAQ, HSPA8, MBP, MSN, PPP2R1A, SPTAN1, SPTBN1, STXBP1, SUMO3, SYN1, VIM*down-regulated*DPYSL2, GARS, NAPA (includes EG:108124), TCP1*not in the dataset*Actin, Alpha Actinin, Alpha catenin, Alpha tubulin, Beta Tubulin, Calmodulin, Clathrin, Dynamin, F Actin, Hsp27, PLC gamma, Spectrin, Tubulin
Cell Death, Developmental Disorder, Neurological Disease	42	21	*up-regulated*CTNNB1, ENO1, GFAP, GIT1 (includes EG:216963), HBB, HLA-C, PSMD2, PTPRZ1, SOD1, STAT1, STAT2, TAPBP, TRRAP*down-regulated*APP, CSNK2A1, FASN, LRP1 (includes EG:16971), PELP1, PRKACB, SFPQ, SUGT1*not in the dataset*26s Proteasome, Akt, Cyclin A, Cyclin E, ERK1/2, Focal adhesion kinase, Histone h4, Hsp70, Hsp90, MHC Class I (complex), MHC Class II (complex), NFkB (complex), RNA polymerase II, Secretase gamma
Molecular Transport, Cellular Function and Maintenance, Nucleic Acid Metabolism	38	19	*up-regulated*ALB, ALDOA, API5, APOA1, ARPC5, ATP1A1, ATP1B1, ATP2B2, ATP5B, CAPN9, HEPACAM, HP, HPX, METAP2, OPCML, TF, WDR72*down-regulated*Cyfip2, TANC2*not in the dataset*AGT, APOC1, APOF, ATP5, ATP synthase, CACNA1A, calpain, CD163, CHD1L, FXYD2, HDL, hemoglobin, HTT, miR-340-5p (miRNAs w/seed UAUAAAG), miR-488-3p (and other miRNAs w/seed UGAAAGG), RBP4
***Late vs Early***			
Cell Morphology, Cellular Assembly and Organization, Cellular Function and Maintenance	40	19	*up-regulated*ACTB, ARL8B, CAMK2A, CDK5, CTNNB1, CTTN, DNM1, HSPA8, TUBB, WASF1*down-regulated*APP, CRMP1, DPYSL2, DPYSL3, MAP2, MAP1B, NAPA (includes EG:108124), NCAM1, TCP1*not in the dataset*26s Proteasome, Actin, Beta Tubulin, Calmodulin, Alpha tubulin, CaMKII, CRMP, Dynamin, F Actin, Focal adhesion kinase, Hsp27, Hsp70, Hsp90, PLC gamma, Secretase gamma, Tubulin
Organismal Injury and Abnormalities, Respiratory Disease, Hematological Disease	38	18	*up-regulated*AHSG, ALB, CAMK4, CSNK2A1, EEF2, GFAP, HBA1/HBA2, HBB, RPS21, STAT1, SUMO3, TAPBP, TF, UBE2V1, VIM*down-regulated*GARS, PRKACB, SFPQ*not in the dataset*Akt, APOA4, Cbp/p300, Ceacam1, Creb, DEFB103A/DEFB103B, DEFB4A/DEFB4B, ELP2, HDL, hemoglobin, Ifi47, IFIT1B, NFkB (complex), RNA polymerase II, SPINK7, Stat1 dimer, UBE2F
Infectious Disease, Endocrine System Development and Function, Energy Production	35	16	*up-regulated*ATP6V1B2, CAD, CBR1, CNNM3, GANAB, HSDL1, KHSRP, RANBP3, RRAGC, VPS45*down-regulated*CAT, ABCF3, ACOT7, CLCN6, HSD17B4, PCM1*not in the dataset*ACIN1, ATP6AP1, GTF2H4, HADH, HSD17B7, HSD17B10, KHK, LAMTOR1, LAMTOR2, LAMTOR3, MOCS2, NOA1, PITPNA, PTP4A2, RRAGB, SLC35E1, TMEM173, TOE1, UBC

### Networks, Biological Pathways and Functions Involved in the Clinical Evolution of WNV Infection at the Brain Level

To understand and highlight the relationship of these differentially regulated proteins and the consequences of these modifications in the context of their cellular function, during the course of WNV infection in the brain, before and after the appearance of clinical symptoms, a bioinformatics analysis using a web-based entry tool developed by Ingenuity Systems, Inc. (http//www.ingenuity.com) was performed. The 148 unique significantly differentially regulated host proteins were uploaded into IPA to statistically determine the functions and pathways that were most strongly associated with the protein list and to establish interactions with other proteins in known networks.

At the early time-point after WNV-infection compared to the mock-group, the mapping of our protein dataset (n = 93) onto biological pathways and disease networks allowed us to identify a total of five relevant networks. Three of these top networks were represented with more than 20 focus molecules involved in functions related to protein synthesis and cell death (network 1), nervous system development and cell morphology (network 2), molecular transport and cell cycle (network 3) (Table 2). Among the 23 differentially regulated proteins from network 1, five proteins (HSP70, HSP90AB1, HSPA8, SUGT1, UBE2V1) that are associated with the ubiquitination pathway were found to be mainly down-regulated, and some of them are linked to the down-regulated ubiquitin-like SUMO3 (Figure S2A). In contrast, an increase of STAT1 and STAT2, proteins that are known to be involved in the activation of the innate antiviral responses of the host, particularly the type I interferon response (*e.g.*, IFN-α, IFN-β), was observed. The differential regulation of these groups of proteins, which have critical roles in many cellular processes, is particularly interesting. Although 198 canonical pathways could be generated at the WNV-E time-point, 30 of them presented a significant association (−Log (p-value) >2.0), as indicated in Table S6. The most relevant pathways were related to entry and exit of the virus including clathrin/caveolar-mediated endocytosis but also to mediators of cytoskeleton organization (Rho protein family and actin cytoskeleton signaling), cell-to-cell interactions (Integrins, FAK signaling), and the protein ubiquitination pathway. In addition, biological functions related to these proteins, ranked by significance, corresponded to cellular assembly and organization (*p*-value: 9.69E-07), cellular function and maintenance (*p*-value: 9.27E-06), and cellular development (*p*-value: 5.90E-05). In terms of diseases and disorders, in addition to 37 molecules associated with neurological disease, which had the strongest *p*-values (2.66E-06), 22 proteins were also significantly associated with inflammatory disease and host response (*p*-value: 1.01E-05), including mostly up-regulated proteins such as ACT, DCTN, DNM1, GFAP and STAT1. Collectively, this analysis indicated an important effect of WNV-infection at the early time-point on (i) host response via down-regulation of ubiquitination-related proteins, (ii) on virus entry via up-regulation of clathrin-mediated endocytosis and (iii) on cell morphology/cytoskeleton network.

At the late-time-point after WNV infection compared to the mock group, the use of IPA identified relationships among the 96 modified proteins and generated a total of five networks. The top three networks contained at least 19 focus molecules that have functions related to cellular assembly and organization, nervous system development, cell death (network 1); cell death and neurological diseases (network 2); and molecular transport and cellular function and maintenance (network 3) (Table 2). Among the most significant biological functions determined by IPA, cell death (*p*-value: 6.95E-11) was the first that was identified and included 52 out of the 96 proteins (54%) that are differentially regulated between the late and mock-infected samples. To provide a greater degree of molecular detail from this biological function, a sub-network of interactions between cell death-related proteins was built *de novo* using IPA (Figure S2B). This sub-network showed that 73% of these proteins with a cell death function interact directly or indirectly with each other. Among them, the down-regulated APP protein seems to play a central role in this network and interact with several of the differentially regulated proteins. The abundance variation of APP following virus infections has been recently reported [35]. In addition, 30 molecules were found to be significantly associated with inflammatory disease and response (*p*-value: 6.97E-11), and several of them were associated with the cell death sub-network. In addition to their association with inflammation, the analysis revealed that most of these “cell death” proteins were also related to neurological disorders. Interestingly, 22 of the proteins from the cell death sub-network were not found to be differentially regulated at the early time-point, suggesting that the clinical onset could be linked to CNS cell degradation. Between the late and mock-infected samples, 236 canonical pathways were generated, among which 23 presented a significant association (−Log (*p*-value) >2.0, Table S7). The most relevant pathways were related to entry of the virus including clathrin/caveolar-mediated endocytosis, as observed at the early time-point, neurological disorders, and the protein ubiquitination pathway.

Considering the 65 differentially regulated molecules between the late and early time-points, the IPA system generated four networks. The top three networks (Table 2) contained at least 16 focus proteins that have functions related to cell morphology, cell assembly and organization and cell function and maintenance (network 1); organismal injury and abnormalities, respiratory disease, and hematological disease (network 2); and infectious disease, endocrine system development and function, and energy production (network 3). The highest scoring network was composed of 19 proteins from our dataset and mainly contained up-regulated proteins that are related to cytoskeleton organization, such as actin, tubulin and dynamin, and down-regulated proteins that are related to nervous system development, including APP, DPYSL proteins, MAP1B and MAP2, the latter of which is associated with neurogenesis and axonogenesis. In addition, among the most significant functions and diseases listed by IPA, 36 proteins were associated with cell death and 34 with neurological disease, representing more than half of the proteins. The analysis also generated 214 canonical pathways among which 16 were highly significant (−Log (*p*-value) >2.0, Table S8). The first of these relevant pathways was related to clathrin-mediated endocytosis, as was observed for the comparisons of the early and late time-points with the experimental group. These data highlighted that this pathway appears to be particularly perturbed during the course of WNV infection. Among the other canonical pathways that were generated by IPA, several reflect neurological disorders such as the alteration of proteins involved in amyloid processing, neuronal semaphorin signaling, cell junction signaling or synaptic potentiation. Taken together, the analysis of proteins that are differentially regulated at the late stage of infection pointed to their involvement, in addition to viral circulation, in processes that are linked to neurological disorders and nervous system development impairment, eventually leading to cell death.

### Verification of Protein Abundance Variations from Selected Candidates

To verify the 2D DIGE and iTRAQ results, WB analyses were performed. Among the 148 differentially regulated proteins identified, a total of 12 candidates were selected as being representative of the altered of the cytoskeleton organization (DNM1, VIM, MAP1B, MAP2, CLTC), the ubiquitination pathway (HUWE1), the inflammatory response (STAT1 and phospho-701 STAT1, STAT2, PRDX6) or the nervous system and the cell death pathway (GFAP, CAPN9), according to the availability of the corresponding antibodies. For all WB, each protein sample was labeled with cyanine-3 dye to reveal the variations in sample loading that were taken into account for the normalization and the calculation of the average band volume ratio that was detected by each selected antibody and revealed by fluorescence-conjugated secondary antibody (FITC or ECL Plex system). In these conditions, a precise determination of the protein abundance according to the course of WNV mouse brain infection was performed.

**Figure 4:**
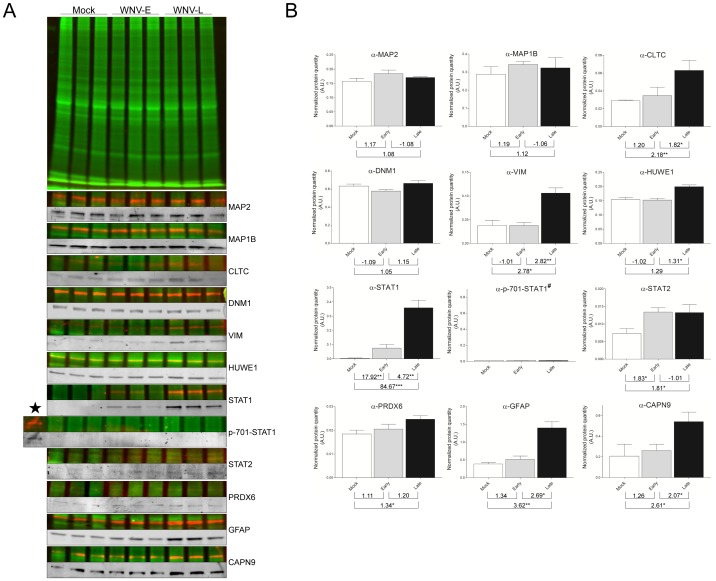
Western blot validations of differentially regulated proteins identified by 2D-DIGE and/or iTRAQ analyses. (A) Protein samples from each group used for proteomic analysis were minimally labeled with cyanine-3 dye. At the top, a representative protein profile of three biological replicates from brain lysates of mock-, and WNV early- and late-infected mice, separated by 10% SDS-PAGE is shown. WB with fluorescence-based methods was used to detect an overlaid fluorescent scan of the general protein patterns (Cy3 dye (green)) and the specific immunoreactive proteins (FITC or Cy5 dye (red)). To better visualize protein detection signals observed with each specific antibody used, corresponding cropped WB images are presented in grey levels. (B) The graphs correspond to the mean ± S.D. of protein quantity measured by densitometry of the antigenic bands. Densitometry analyses were performed using TotalLab Quant v12.2 software (Nonlinear Dynamics), and data were normalized to levels of global protein pattern intensity. The values indicated under each graph correspond to fold changes from paired comparisons (*i.e.,* WNV-E/mock, WNV-L/mock, WNV-L/WNV-E). WNV-E/−L, biological replicates of brain samples infected by West Nile Virus and collected at early or late time-points. The significance of the differential protein expression are indicated *, p<0.05; **, p<0.01; ***, p<0.001. A.U., arbitrary units. α-, antibody anti-; ★, IFN-γ activated mice bone marrow derived macrophage (p-701-STAT1 positive control); #, no quantification for p-701-STAT1. CAPN9, calpain 9; CLTC, clathrin heavy chain; DNM1, dynamin 1; GFAP, glial fibrillary acidic protein; HUWE1; E3 ubiquitin-protein ligase; MAP1B, microtubule-associated protein 1B; MAP2, microtubule-associated protein 2; PRDX6, peroxiredoxin 6; STAT1/2, signal transducer and activator of transcription 1/2; VIM, vimentin.

For proteins that are involved in cytoskeleton organization, the significant up-regulation of VIM at the late time-point was confirmed. The transitory increased abundance of microtubule-associated proteins (MAP2 and MAP1B) at the early time-point was observed by WB experiments. However, statistical analysis did not succeed to validate these transitory protein abundance increases. The lower protein expression variations observed by WB than those detected by proteomic approaches (Figure 4, Table S5), and the intra-group variations (*i.e.,* standard deviations) between the different biological replicates, of protein quantity measured for some time point, are factors which could alter these validation steps. Concerning CLTC and DNM1 proteins that were both found with increased protein abundance during the time course of WNV infection (Tables 1, S4 and S5), the confirmation of the significant up-regulation was only successfully obtained for CLTC (Figure 4). The absence of the significant variation in the level of DNM1 by WB could be attributed to the presence of DNM1 isoforms. Effectively, DNM1 is known to undergo post-translational modifications (*i.e.,* extensive rounds of phosphorylation and dephosphorylation), leading to several isoforms with different isoelectric points that are involved in the activation of the CME pathway [36]. Although the abundance of several DNM1 protein spots were determined to be altered by 2D-DIGE (Tables 1 and 2, Figures 2 and S1), it is possible that the amount of altered and unaltered spots merged in the same 1D protein band and thus did not significantly affect the total level of DNM1 protein detected by 1D WB. Complementary experiments investigating changes in the DNM1 phosphorylation state according to the host clinical evolution following WNV infection are necessary to clarify the mechanism of protein regulation. For proteins that are involved in the protein ubiquitination pathway, the kinetic down-regulation of HUWE1 during WNV infection, as determined by iTRAQ analysis, could not be confirmed by WB, and in contrast a significant protein abundance increase was detected between late and early time points.

For proteins that are related to the inflammatory response, the kinetic augmentation of STAT1 protein during WNV infection (Table S5) was statistically confirmed using a specific antibody. Conversely, in control samples, these proteins were found at a very low or undetectable level (Figure 4). Despite the low signal detected by WB against STAT2, the increase of STAT2 abundance was confirmed statistically at the early and late time-points compared to the mock condition. As the phosphorylation of STAT proteins is required to obtain a functional Jak/STAT signaling pathway [37], analysis of their phosphorylation states was performed. Using p-701-STAT1 antibodies, STAT1 phosphorylation was detected only in the positive control sample (*i.e.,* murine macrophages activated with IFN-γ). Whereas STAT1 protein abundance dramatically increases at both time-points compared to mock, p-701-STAT1 was not detected, suggesting an inhibition of the Jak/STAT signaling pathway by WNV. Despite the modest variation of PRDX6 level, the increased abundance of this protein was significant solely at the late time-point compared to mock, as determined by 2D-DIGE analysis (Table 1).

For proteins involved in nervous system development and cell death, the progressive up-regulation of GFAP during the course of WNV infection was detected by WB, but this protein augmentation was found statistically significant only at late time point. Concerning CAPN9, the increase of protein quantity was statistically confirmed in the late samples.

Collectively, the abundance variations of the majority of the selected protein candidates were validated by WB while taking into account the kinetic clinical mouse symptoms (*i.e.,* before and after the appearance of neurological disorders corresponding to the early and late time-points). The inability to validate the protein-level variation for some of these proteins could be attributed to the presence of unmodified isoforms that could not be distinguished by 1D WB, to the low expression fold-change determined by proteomic approaches or by the low number of biological replicates, which may be difficult to confirm despite this rigorous and original quantitative WB method. Further experiments using more discriminate quantitative methods, such as selected reaction monitoring mass spectrometry, would be needed to support some of these unconfirmed protein abundance variations [38].

## Discussion

The current study analyzed the kinetic changes of protein expression in mouse brain tissue samples that were collected at early and late time-points post-viral infection, corresponding to the absence and the appearance of neurological clinical symptoms, respectively, using comprehensive quantitative proteomic approaches. A total of 148 unique proteins were found to be differentially regulated following WNV infection, indicating a profound host proteome modification at the brain level. The bioinformatics analysis (Ingenuity Pathway Analysis) of each protein dataset of the different time-point comparisons revealed that four major functions were altered during the course of WNV-infection in the mouse brains: i) modifications of cytoskeleton maintenance associated with virus circulation; ii) deregulation of the protein ubiquitination pathway; iii) modulation of the inflammatory response; and iv) alteration of neurological development and neuronal cell death. Networks and pathways that are associated with differentially regulated proteins are discussed to characterize the pathophysiologic processes of neuroinvasive WNV infection at the early and late time-points. Interestingly, experiments with null mouse models have evidenced the involvement of the innate and adaptive immune response in controlling WNV neuroinvasion [39], [40], [41]. The absence of white blood cell proteins detection involved in the immune response could likely attributed to their low abundance compared to brain proteins, and then were under the detection limit of the present proteomic approaches. Nevertheless, it seems conceivable that some of the protein differentially regulated could result from the presence of immune cells at the neuroinflammatory foci’s. However, among the proteins differentially regulated, it cannot be excluded that some protein abundance variations were attributed to passive phenomena. Complementary experiments are needed to distinct differentially regulated proteins participating directly to clinical outcomes from that reflecting virus replication.

### i) Modifications of Cytoskeleton Maintenance and Virus Circulation

#### Virus entry via clathrin-mediated endocytosis (CME)

As viruses are obligate intracellular pathogens, viral entry into target cells is necessary to initiate replication and infection. The use of CME for virus entry has been described for several viruses, including influenza [42], hepatitis B [43], orthobunyavirus [44] and WNV [45].

**Figure 5:**
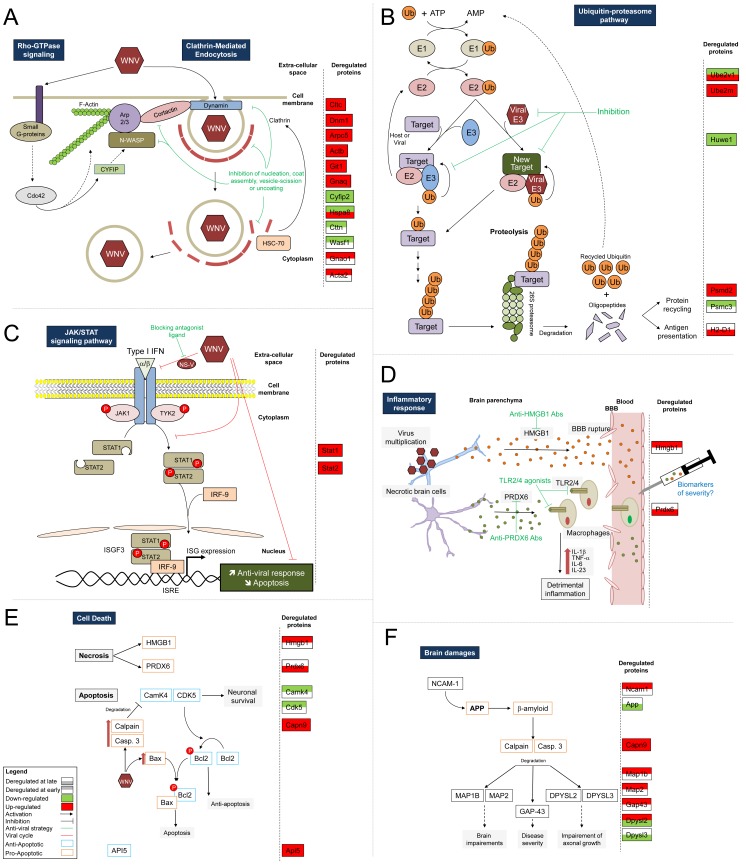
Schematic illustration of key pathways or biological functions altered during the course of WNV infection. Host proteins that were found to be significantly differentially regulated by proteomic approaches were located in relevant pathways based on the IPA database results and a careful reading of published literature. The proteins that were determined to be differentially regulated in our study are indicated in the right portion of each panel, according to the different time-point comparisons. Several functions were altered during the course of WNV-infection in the mice brain, including: (A) The cytoskeleton remodeling associated with virus circulation as evidenced by the WNV hijacking of the clathrin-mediated endocytosis pathway (CME) and Rho GTPase signaling. (B) The perturbations of the protein ubiquitination pathway allowing viral proteins to avoid degradation and/or antigen presentation. (C) The regulation of the JAK/STAT signaling pathway corresponding to an evasion mechanism against activation of the antiviral response. (D) The kinetic modulation of the inflammatory response leading to brain injuries. (E) Cell death comprising neuronal necrosis and apoptotic phenomena. (F) The brain damage reflected by the abundance variation of numerous proteins involved in the deterioration of neurological functions. Known partners of identified differentially regulated molecules are indicated. Anti-viral strategies and potential biomarker candidates associated with the severity of clinical evolution are suggested as indicated in green and blue, respectively. A legend is shown in the bottom left corner. Abbreviations of IPA-uploaded proteins are listed in Tables 1, S4 and S5. The others are listed as follows: Bax, Bcl2-associated X protein; BBB, blood-brain barrier; Bcl2, B-cell lymphoma 2; Casp.3, Caspase 3; Cdc42, cell division cycle 42; E1 and E2, ubiquitin-conjugating enzyme; E3, ubiquitin protein ligase; IFN, interferon; IL, interleukin; IRF9, interferon regulatory factor 9; ISG, interferon-stimulated gene; ISGF3, IFN stimulated gene factor 3; ISRE, interferon-stimulated response element; JAK1, Janus kinase 1; NS-V, non-structural viral protein; TLR, Toll-like receptor; TNF-α, tumor necrosis factor alpha; TYK2, tyrosine kinase 2; Ub, ubiquitin.

Bioinformatic analysis of our datasets revealed that CME was one of the most significant canonical pathways generated by IPA, involving several host proteins that are differentially regulated at both the early and late time-points. Several key factors participating in the CME pathway were identified (Figure 5A). Our data support previous *in vitro* experiments suggesting that WNV uses the CME pathway to gain entry into host cells [45]. Clathrin is recruited for vesicle-coating, dynamin triggers vesicle scission from parent membrane and heat-shock cognate 70 functions as an ATPase in clathrin-coat disassembly [46]. Although actin is dispensable for CME, it could interact with the clathrin network after recruitment of Arp2/3 to the budding vesicle, which is mediated by cortactin and neural Wiskott-Aldrich syndrome protein (N-WASP) [46]. The recruitment of actin polymerization for CME was reported to provide force for plasma membrane invagination and vesicle scission [47], [48], particularly for the uptake of large cargoes such as viruses [49]. Taken together, the up-regulation of these proteins that are required for the CME pathway supports the use of this pathway by WNV *in vivo* to gain host cell entry.

The central role of clathrin function for virus entry into host cells has been previously documented [50], [51]. As WNV entry into host cells occurs throughout the time-course of infection, the development of a strategy to block this critical pathway could be a pertinent approach limiting virus spread prior to and after the development of clinical events, as was recently shown for dengue virus replication *in vitro* [52], [53]. As several other members of the *Flaviridae* family were also shown to enter cells via CME [54], [55], the inhibition of viral endocytosis via the clathrin pathway could be applied for the prevention and/or treatment of a wide range of viral infections [50], [56], [57].

#### Actin cytoskeleton remodeling via Rho GTPase signaling

It appears that the endocytic pathway for WNV is closely associated with the host cell cytoskeleton network. Indeed, to optimize viral replication and virion production, actin cytoskeleton and Rho-family GTPases signaling have been described to be hijacked by many viruses [58]. In this study, the abundance of several proteins related to the actin cytoskeleton and Rho GTPase signaling was found to be modified during the course of neuroinvasive WNV infection (Figure 5A).

Rho-family GTPases control signal transduction pathways linking membrane receptors to the cytoskeleton [58]. Rho GTPases are key regulators of actin and microtubule assembly, are responsible for cell polarity and adhesion, and play an important role in neuronal development [58], [59]. Among the proteins participating in the Rho GTPase signaling pathway, the Arp2/3 complex is a powerful machine for actin filament formation [60]. This complex is activated by cortactin (CCTN) and Wiskott-Aldrich syndrome family proteins (WASP) whose activity is regulated by Rho GTPases such as Cdc42 [61], [62]. Rho GTPases are themselves, under the control of small G proteins that were found to be up-regulated following WNV infection (GNAO1 and GNAQ). The augmentation of small G proteins at the early time-point could trigger Rho GTPase signaling, which leads to significant actin rearrangements [63].

Interestingly, the initial interaction of herpes virus with the cell surface could induce Rho GTPase signaling, leading to significant actin rearrangements [63], [64]. This interaction was shown to facilitate the successful delivery of viral particles into the host cell cytoplasm. Based on our results and previous studies, we can hypothesize that after binding of a ligand to the G protein-coupled receptor, small G proteins are the starting point of cascades of activation of Rho GTPases such as Cdc42 proteins, leading to the activation of Arp2/3 by different effectors and thus inducing the regulation of actin polymerization (Figure 5A). The up-regulation of numerous proteins involved in these pathways at the early time-point indicates a strong alteration of the cytoskeleton organization after WNV infection.

At the late time-point, several of the aforementioned proteins remained differentially regulated (*e.g.,* ACTB, ARPC5, GNAQ, GIT1, GFAP and CYFIP2) and an increased level of other proteins that are involved in cytoskeleton maintenance, such as myosin (MYO18A), moesin (MSN), spectrin (SPTBN1, SPNA2), vimentin (VIM) and syntaxin (STXBP1), were detected. These multifunctional proteins are involved in the regulation of cell morphology, plasma membrane stability and synaptic vesicles trafficking [65]. The abundance variation of some of these proteins has already been shown following other viral infections [66], [67], [68], [69], [70]. Although their exact role following WNV infection remains to be analyzed in detail, the increased abundance of these cytoskeletal proteins at the late time-point may participate in viral particle assembly, cargo and egress leading to the replication and release of mature virions. Taken together, these differentially regulated proteins found in our study show that WNV infection strongly alters the cytoskeleton organization and dynamics, particularly via Rho GTPase signaling. The role of molecules from the Rho GTPase signaling pathway in cytoskeleton rearrangement has been reported to promote the entry, replication and spread of many viruses [58], and the inhibition of Rho GTPase signaling in African Swine Fever Virus (ASFV)-infected cells has been shown to decrease viral replication [71].

### ii) Involvement of Protein Ubiquitination Pathway

Recently, many viruses have been reported to utilize or manipulate the host ubiquitin-proteasome pathway (UPP) to escape the cellular immune response, to facilitate virus replication and to promote virus assembly and budding [72]. We found nine differentially regulated proteins that are involved in UPP following WNV-infection. Among them, some proteins were differentially regulated at both time-points in the same direction (UBE2M, PSMD2, SUGT1) or in the opposite direction (HSP8, UBE2V1) or differentially regulated only at one time-point (at the early time-point: HSP90AB, PSMC3; at the late time-point: HSPA12A, HLA-C). At the early time-point, the abundance of the differentially regulated protein from the UPP mainly decreased, while at the late time-point, an up-regulation of several of these proteins was observed. The late time-point up-regulation of E2 enzymes (UBE2M and UBE2V1), that are responsible for the conjugation of ubiquitin to protein substrates, and of MHC class I proteins (HLA-C) could represent a host response to constrain viral development by an augmentation of foreign antigen presentation (Figure 5B). Although the alteration of protein expression from the UPP seems to be a common phenomenon following virus infection [72], the use of specific drugs against proteins from UPP could be an efficient antiviral strategy. UPP inhibitors target proteasomal functions leading to a complete inhibition of the ubiquitin pathway [73]. The development of drugs targeting E ubiquitin-protein ligases, which usually determine substrate specificity, could inhibit specific ubiquitin-substrate binding, maintaining the other UPP degradation functions [74].

### iii) Activation/Repression of the Inflammatory Response

The activation of interferon type I pathways (*e.g.*, IFN-α, IFN-β) represents a major antiviral defense mechanism limiting virus levels, reducing neuronal death, and increasing cell survival [75]. To ensure their survival in the infected host, several viruses have developed distinct strategies to block the Jak-STAT signaling pathway, either by inhibiting Jak phosphorylation [76], [77], by inducing the degradation [78], or by blocking the phosphorylation of STAT1 and/or STAT2 proteins, preventing their nuclear translocation (Figure 5C) [79]. In the present study, a significant augmentation of STAT1 and STAT2 protein levels was detected at the early time-point and was maintained at the late time-point. The augmentation of STATs proteins could correspond to a host response following WNV infection, in agreement with previous findings [80], [81]. However, the increase of STAT protein abundance was not correlated with an increase of STAT phosphorylation states, as confirmed by WB using a p-701-STAT-1-specific antibody. Thus, the augmentation of STAT1 and STAT2 should be linked to the inhibition of STAT phosphorylation, blocking their nuclear translocation. Moreover, previous *in vitro* studies have reported that non-structural proteins from WNV can act as IFN antagonists, leading to the inhibition of STAT1 and STAT2 phosphorylation and their subsequent translocation to the nucleus [82], [83], [84]. As blocking IFN signaling appears to be an obvious strategy for successful virus infection, the restoration of the Jak-STAT pathway by inhibiting viral protein binding to IFN receptors or effectors could be a strategy to extend antiviral therapeutics.

Among other molecules involved in the inflammatory response, high mobility group box 1 (HMGB1) and peroxiredoxin (PRDX6), are up-regulated at the early and late time-points, respectively and have been classified as damage-associated molecular patterns (DAMPs) [85]. HMGB1 and PRDX6 proteins that are released from necrotic brain cells are able, respectively, to induce blood brain barrier (BBB) disruption and bind to macrophage toll-like receptors (*i.e.,* TLR2 and TLR4), inducing the production of inflammatory cytokines (*i.e.,* IL1β, TNFα, IL6 and IL23) [86], [87]. The production of these cytokines has been described in cell culture highlighting the strong inflammatory response after WNV infection that leads to neuronal death [88]. Thus, the increased expression of HMGB1 and PRDX6 could be effectors of detrimental inflammation in the brain of WNV-infected mice and brain injury following WNV infection (Figure 5D).

Interestingly, a delay in the production of HMGB1 and PRDX in response to brain ischemic stroke was reported with HMGB1 being released prior to PRDX6 [89]. This delayed expression, which was also observed in the present study, suggests that WNV infection-induced encephalitis may result from similar inflammatory events as seen in ischemic stroke injury. In addition, the plasma level of HMGB1 was proposed as a predictive marker of traumatic brain injury [90] and the disease outcome of dengue virus infection [91], [92]. In addition, the administration of anti-HMGB1 neutralizing antibodies has been shown to preserve BBB integrity [93] and suppress the expression of inflammatory molecules such as TNF-α in the case of brain injury [94]. A beneficial effect of the use of anti-PRDX antibodies was also reported, with the reduction of inflammatory cytokines (*i.e.,* IL23 and IL17) [89]. Thus, the investigation of the release of HMGB1 and PRDX6 could be useful to evaluate the degree of brain injury following WNV infection. Moreover, in the scope of a therapeutic strategy, the injection of specific antibodies targeting HMGB1 and/or PRDX6 could have a protective effect against brain alteration, limiting BBB disruption, and on macrophage recruitment and stimulation [89]. In addition to the inhibition of these molecules, blocking the receptors (*i.e.,* TLR2, TLR4) or acting on the downstream cytokines that are produced (*i.e.,* IL23, IL17), could be alternative ways to reduce the inflammatory response that is induced by WNV infection [86], [87].

### iv) Activation of Neuronal Cell Death and Alteration of the Nervous System

Replication of WNV in the brain has been repeatedly described to induce neuronal injury, leading to neuronal cell death [95], [96], [97]. Here, more than 40% (n = 38) and 55% (n = 53) of the proteins that are significantly differentially regulated at the early and late time-points, respectively, compared to the mock-infected group, were involved in cell death according to IPA, underlining the large alteration of this biological function during the course of neuroinvasive WNV.

Among them, at the early time-point, the protein level of CamK4 and CDK5, two kinases that are involved in the inhibition of apoptosis, decreased (Figure 5E) [98], [99]. To promote its anti-apoptotic role, CDK5 phosphorylates Bcl2 [100]. However, an up-regulation of Bax has been reported to be associated with WNV-induced apoptosis [101]; Bax can bind Bcl2 to suppress its anti-apoptotic function [102]. Moreover, an inhibition of CamK4 has been shown to induce neuronal cell death [103]; this kinase can be cleaved by caspase-3 or calpain [104], and the up-regulation of calpain-9 protein (CAPN9) may then participate in the decreased protein level of CamK4. Taken together, the diminution of anti-apoptotic factors (*i.e.,* CDK5 and CamK4) and the increase of calpain, a pro-apoptotic factor, suggest that the environment seems favorable for cell death induction following WNV infection. Nevertheless, the increased abundance of the apoptotic inhibitor protein 5 (API5), at both time-points, indicates that the mechanism of cell death regulation is particularly complex. Effectively, a premature cell death could represent a host defense mechanism that limits viral replication; in contrast, intracellular virus replication could also lead to cytopathic effects and cell death [105]. The up-regulation of anti-apoptosis factors could enhance the survival of WNV-infected host cells, contributing to viral persistence and replication. Complementary experiments would then be needed to determine whether the differential regulation of proteins involved in cell death corresponds to a host response that is designed to eliminate the virus, or conversely attributed to virus replication.

WNV infection-induced brain damage is supported in our *in vivo* model by the abundance variation of numerous proteins that have been previously described as markers of central alteration (Figure 5F).

NCAM-1 predominantly expressed in neurons and glial cells plays an important role in neuronal development and, in particular, the formation and the development of axons, dendrites and synapse plasticity [106]. In this study, NCAM-1 was found to be up-regulated at the early time-point. NCAMs have been used as markers for neurological disorders such as multiple sclerosis or encephalitis [107], [108]. To our knowledge, this is the first time that an alteration of NCAM-1 protein expression was reported following WNV infection.

GAP43, a marker of axonal growth that is involved in synapse plasticity, was up-regulated at the early time-point. GAP43 expression was shown to be inversely associated with the intensity of neuronal injuries in mice (*i.e.,* increased and decreased expression following moderate and severe brain injury, respectively) [109]. Here, the GAP-43 expression profile suggests that GAP-43 could activate brain repair mechanisms at the early time-point, which diminished according to the severity of injuries. As GAP-43 protein integrity is under the control of calpain [110], treatment with calpain inhibitors could enhance neuronal repair mechanisms and neuroprotective effects.

At the late time-point, the abundance of APP, DPYSL2 and DPYSL3 decreased. APP is a transmembrane glycoprotein that may have a key role in nervous system development through its involvement in synapse formation, neuronal migration and motility [111], [112]. APP is a central component of Alzheimer’s disease (AD), where its cleavage generates β-amyloid peptides. The decrease in APP abundance at the late stage could be a result of its proteolysis, leading to an augmentation of β-amyloid peptides. This hypothesis is in concurrence with a previous proteomic study performed on WNV-infected neurons that reported an up-regulation of β-amyloid peptide [26].

DPYSL proteins (or CRMPs) are mediators of the Sema3A signaling involved in axon and dendrite growth and guidance [113], [114]. Interestingly, DPYSL2 was found to be up-regulated at the early time-point and further showed a decreased level of protein abundance at the late time-point together with DPYSL3. The diminution of DPYSL2 has previously been observed in rat neurons infected *in vitro* with WNV using a proteomic approach [26]. DPYSL proteins can also be cleaved by calpain in neurological damage [115], [116]. Thus, calpain, with its links to APP, which was located at the center of the sub-network generated by IPA that was restricted to proteins related to cell death at the late time-point, appear to be the critical molecules involved in neuronal death following WNV infection.

The differential regulation of glial- (*i.e.,* GFAP) and oligodendrocyte- (*i.e.,* MBP) specific proteins indicated that *in vivo* WNV infection affects major brain cell types. GFAP, the main intermediate filament (IF) protein in mature astrocytes, was found to be up-regulated during the entire time-course of WNV-infected mice, and was statistically validated by WB at late time point. GFAP has been repeatedly mentioned as a reliable biomarker of brain injury that is released into the CSF and blood [117], [118]. In CSF from patients with WNV meningoencephalitis, an increase of GFAP was noted; however, the detection of this protein in individuals presenting only WNV fever underlined that glial brain degradation could occur in the absence of severe clinical symptoms [119], [120]. Finally, GFAP could represent a biomarker candidate of brain injury and central virus affect.

Myelin basic protein (MBP) is a major constituent of the myelin sheath of oligodendrocytes in the CNS, and it has been reported as both a central (*i.e.,* CSF) and peripheral (*i.e.,* sera) marker of brain myelinated axonal damage [121]. Elevated serum levels of MBP have been observed in ischemic stroke [122] and autoimmune diseases such as multiple sclerosis [123], [124], reflecting BBB disruption and demyelination process. The increased expression of MBP at the late time-point in our study could correspond to repair mechanisms following myelin sheath degradation. *In vitro* experiments have previously demonstrated that oligodendrocytes were susceptible to WNV infections [125], [126]. Brain demyelination has been previously reported in humans and rodent models infected by encephalitic viruses such as HIV [127], Theiler’s Murine Encephalitis Virus [128], or Japanese encephalitis virus (JEV) [129]. In JEV-infected mice, the T-cell response against MBP increased and this autoimmune inflammatory response seemed to be responsible for axon demyelination [129]. Complementary experiments could be performed to determine whether myelin destruction occurred *in vivo* during WNV-infection and whether this myelin degradation may be attributed to an autoimmune phenotype.

### Conclusion

Due to their limited coding capacity, viruses hijack proteins and pathways of the host to replicate. The combination of comprehensive quantitative proteomic approaches with bioinformatics analysis revealed a wide range of biological processes that were found to be modified during the time course of WNV infection in an animal model. Although several of these pathways have been previously described following WNV infection, our study provides novel insight into understanding the host response to WNV infection. The present work unravels crucial virus-host interactions that take place during a severe neuroinvasive disease, identifies putative mechanisms involved in WNV pathogenesis, and offers the description of specific markers of brain cell damage. Finally, the evolution in protein expression profiles uncovered according to clinical symptoms could contribute to the development of new anti-viral therapeutic targets, the identification of additional biomarkers of disease progression, and eventually could improve the diagnosis and monitoring of neurological insults to reduce mortality and neurological sequelae in humans.

## Supporting Information

Figure S1 **2D-DIGE analysis (pH 4–7) of mock- and early WNV-infected brain samples.** Representative data from a 2D-DIGE experiment using a 10% SDS-polyacrylamide gel with the pH range from 4 to 7 are shown. Proteins from mock- and early- WNV-infected brain samples were labeled with Cy3 and Cy5 cyanine dyes, respectively. As determined by Progenesis SameSpot software, protein spots that were differentially regulated between the two experimental conditions (|ratio| ≥1.3 and *p*≤0.05) were submitted to mass spectrometry for identification. The numbers annotated on the gel correspond to master gel numbers of differentially regulated protein spots. All spots were identified as *Mus musculus* and are listed in Table S4. Spots that were differentially modified between WNV-early and mock (B) infected samples are represented by red (up-regulated) or blue (down-regulated) dots.
(TIF)

Figure S2 **Most significant protein networks of differentially regulated proteins following WNV infections.** (A) The first significant network of protein differentially regulated between WNV-E and mock-infected mice. Network 1 that was associated with “protein synthesis and cell death” was generated by Ingenuity Pathway Analysis (IPA) software using the list of differentially expressed proteins at the early time-point following WNV-infection, determined after 2D-DIGE and iTRAQ analyses. (B) Sub-network of cell death-related proteins built using IPA *de novo* between WNV-L and mock-infected mice. Individual proteins are represented as nodes colored in red and green corresponding to up- and down-regulated proteins, respectively, while the nodes (proteins) in white have been added by IPA to maximize the network connectivity. The edges with arrowheads describe the direct (continuous lines) and indirect (dotted lines) nature of the interaction between these proteins. The different shapes of the nodes represent functional classification of the proteins as indicated in the legend.
(TIF)

Table S1 Experimental design for the 2D-DIGE analysis using pH 3–10 IEF.
(DOC)

Table S2 Experimental design for the 2D-DIGE analysis using pH 4–7 or 6–11 IEF.
(DOC)

Table S3 Experimental design for iTRAQ reagent-labeling of brain sample pools.
(DOC)

Table S4 Proteins identified from the differential 2D DIGE analysis (pH 4–7) after WNV infection.
(DOCX)

Table S5 Set of proteins identified by iTRAQ labeling and tandem mass spectrometry as differentially expressed between mock-, early- and late WNV-infected samples, indicating fold-changes and *p*-values in each comparison, and GO subcellular location and biological function.
(DOC)

Table S6 Ingenuity canonical pathways showing a significant association using the set of proteins that are differentially expressed between early- and mock-WNV infected samples [−Log(*p*-value) >2.0].
(DOCX)

Table S7 Ingenuity canonical pathways showing a significant association using the set of proteins that are differentially expressed between late- and mock-WNV infected samples [−Log(*p*-value) >2.0].
(DOCX)

Table S8 Ingenuity canonical pathways showing a significant association using the dataset of proteins that are differentially expressed between late- and early-WNV infected samples [−Log(*p*-value) >2.0].
(DOCX)
