# Correction: Altered Protein Networks and Cellular Pathways in Severe West Nile Disease in Mice

**DOI:** 10.1371/annotation/a01d68f4-f23d-4c0a-a0f8-f32432b0efa7

**Published:** 2013-12-03

**Authors:** Christophe Fraisier, Luc Camoin, Stephanie M. Lim, Mahfoud Bakli, Maya Belghazi, Patrick Fourquet, Samuel Granjeaud, Ab D. M. E. Osterhaus, Penelope Koraka, Byron Martina, Lionel Almeras

The name of the third author is incorrect. The correct name is: Stephanie M. Lim. The correct Citation is: Fraisier C, Camoin L, Lim SM, Bakli M, Belghazi M, et al. (2013) Altered Protein Networks and Cellular Pathways in Severe West Nile Disease in Mice. PLoS ONE 8(7): e68318. doi:10.1371/journal.pone.0068318. The correct abbreviation in the Author Contributions Statement is: SML. 

