# Supplementary figures and images for: Altered Protein Networks and Cellular Pathways in Severe West Nile Disease in Mice
Source: PLoS One. 2013 Jul 10;8(7):e68318. doi: 10.1371/journal.pone.0068318 (PMC3707916; doi:10.1371/journal.pone.0068318)

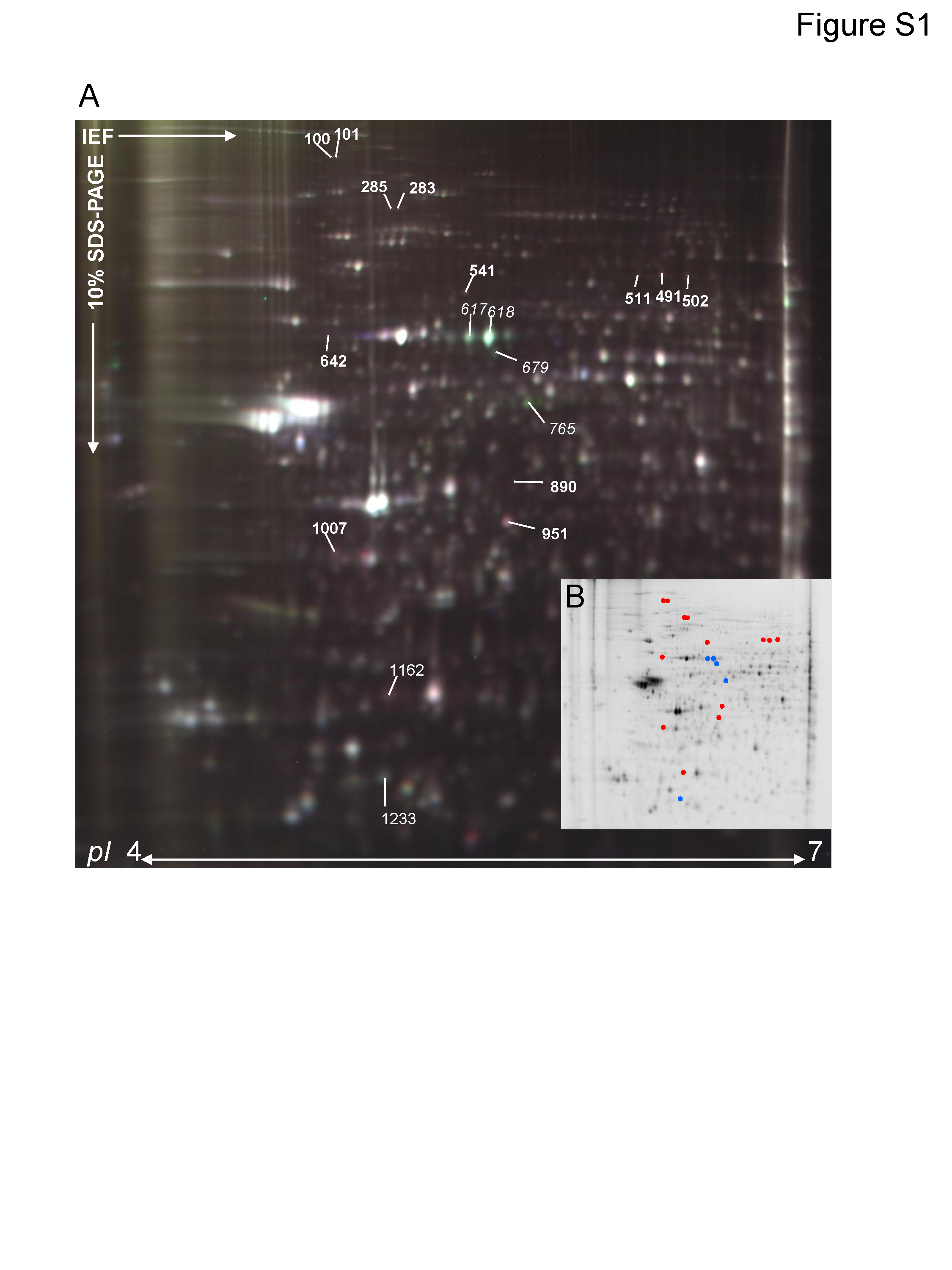

Supplement: Figure S1 — 2D-DIGE analysis (pH 4–7) of mock- and early WNV-infected brain samples. Representative data from a 2D-DIGE experiment using a 10% SDS-polyacrylamide gel with the pH range from 4 to 7 are shown. Proteins from mock- and early- WNV-infected brain samples were labeled with Cy3 and Cy5 cyanine dyes, respectively. As determined by Progenesis SameSpot software, protein spots that were differentially regulated between the two experimental conditions (|ratio| ≥1.3 and p≤0.05) were submitted to mass spectrometry for identification. The numbers annotated on the gel correspond to master gel numbers of differentially regulated protein spots. All spots were identified as Mus musculus and are listed in Table S4. Spots that were differentially modified between WNV-early and mock (B) infected samples are represented by red (up-regulated) or blue (down-regulated) dots. (TIF) [file pone.0068318.s001.tif]

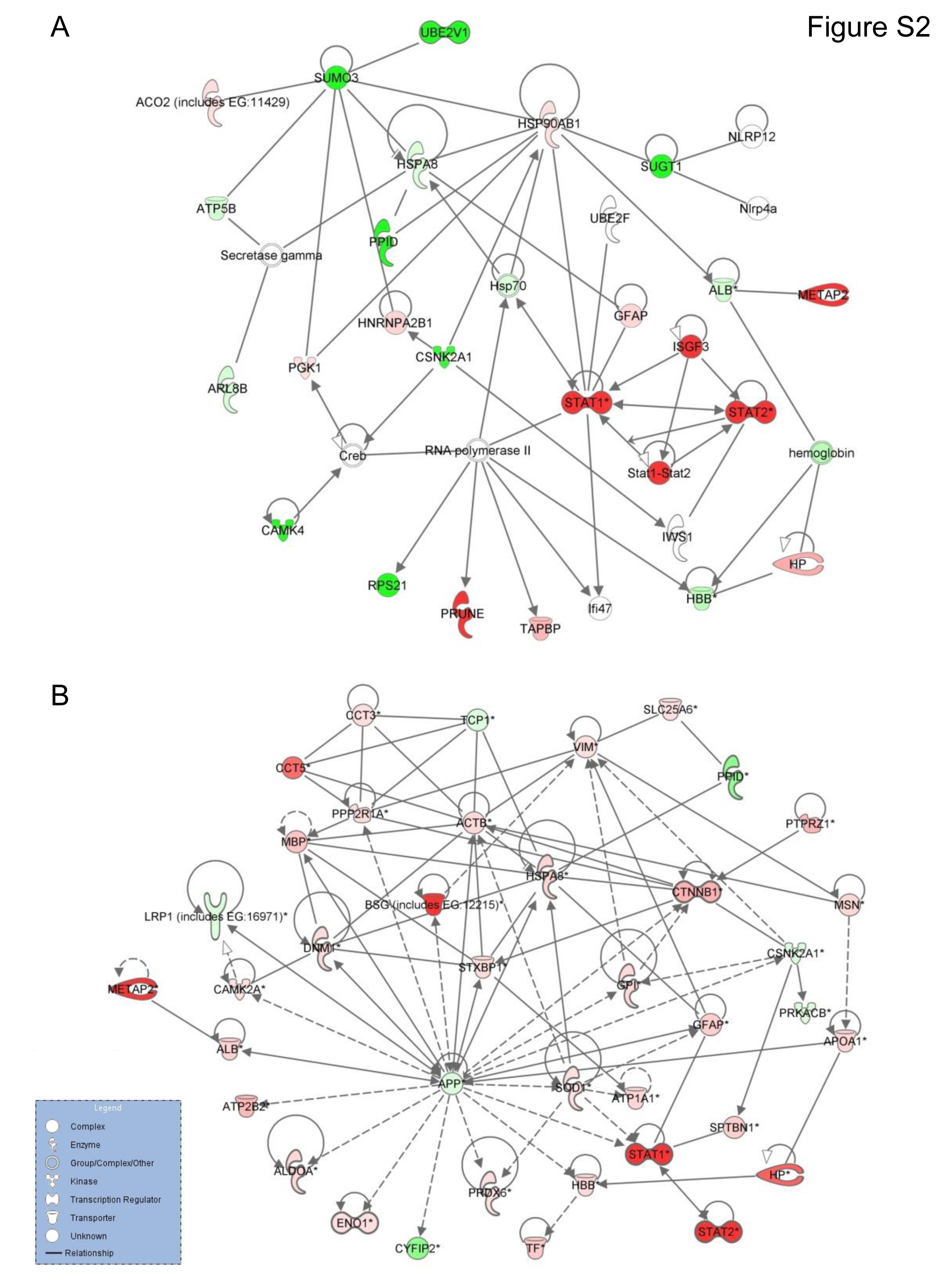

Supplement: Figure S2 — Most significant protein networks of differentially regulated proteins following WNV infections. (A) The first significant network of protein differentially regulated between WNV-E and mock-infected mice. Network 1 that was associated with “protein synthesis and cell death” was generated by Ingenuity Pathway Analysis (IPA) software using the list of differentially expressed proteins at the early time-point following WNV-infection, determined after 2D-DIGE and iTRAQ analyses. (B) Sub-network of cell death-related proteins built using IPA de novo between WNV-L and mock-infected mice. Individual proteins are represented as nodes colored in red and green corresponding to up- and down-regulated proteins, respectively, while the nodes (proteins) in white have been added by IPA to maximize the network connectivity. The edges with arrowheads describe the direct (continuous lines) and indirect (dotted lines) nature of the interaction between these proteins. The different shapes of the nodes represent functional classification of the proteins as indicated in the legend. (TIF) [file pone.0068318.s002.tif]
