# Supplementary material for: Altered Protein Networks and Cellular Pathways in Severe West Nile Disease in Mice
Source: PLoS One. 2013 Jul 10;8(7):e68318. doi: 10.1371/journal.pone.0068318 (PMC3707916; doi:10.1371/journal.pone.0068318)
Supplement: Table S1 — Experimental design for the 2D-DIGE analysis using pH 3–10 IEF. (DOC) [file pone.0068318.s003.doc]

**Table S1.** Experimental design for the 2D-DIGE analysis using pH 3-10 IEF. Mock- (control), early (E) and Late (L) WNV-infected mice brain samples were labelled with cyanine 3 (Cy3) or cyanine 5 (Cy5). An internal standard pool was generated by combining equal amounts of each sample tested in this study, and labelled with Cy2.

| **Gel number** | **Uninfected mice**  **(control=C)** | **Mice infected with *WNV* and**  **brain collected at day2**  **(early=E)** | **Mice infected with *WNV* and**  **brain collected at day5**  **(late=L)** |
| --- | --- | --- | --- |
| 1 | Cy5-C1 |  | Cy3-WN-L1 |
| 2 | Cy3-C3 |  | Cy5-WN-L3 |
| 3 | Cy5-C2 | Cy3-WN-E1 |  |
| 4 | Cy3-C4 | Cy5-WN-E3 |  |
| 5 |  | Cy3-WN-E2 | Cy5-WN-L2 |
| 6 |  | Cy5-WN-E4 | Cy3-WN-L4 |
| 7 | Cy3-C5 |  | Cy5-WN-L5 |
| 8 | Cy5-C6 | Cy3-WN-E5 |  |
| 9 |  | Cy5-WN-E6 | Cy3-WN-L6 |
