# Supplementary material for: Altered Protein Networks and Cellular Pathways in Severe West Nile Disease in Mice
Source: PLoS One. 2013 Jul 10;8(7):e68318. doi: 10.1371/journal.pone.0068318 (PMC3707916; doi:10.1371/journal.pone.0068318)
Supplement: Table S3 — Experimental design for iTRAQ reagent-labeling of brain sample pools. (DOC) [file pone.0068318.s005.doc]

**Table S3.** Experimental design for iTRAQ reagent-labelling of brain sample pools. One hundred microgram of each pooled Mock-WNV infected (C1 to C4), WNV-infected and collected at early time point (WN-E1 and E2) or at late time point (WN-L1 and L2) were digested with trypsin and the resulting peptides of each sample were specifically labelled with one iTRAQ reagent as indicated below, previously to mix all samples.

| **Sample pools** | **Isobaric iTRAQ**  **reagent** |
| --- | --- |
| C1 | 113 |
| C2 | 114 |
| C3 | 115 |
| C4 | 116 |
| WN-E1 | 117 |
| WN-E2 | 118 |
| WN-L1 | 119 |
| WN-L2 | 120 |
