# Supplementary material for: Altered Protein Networks and Cellular Pathways in Severe West Nile Disease in Mice
Source: PLoS One. 2013 Jul 10;8(7):e68318. doi: 10.1371/journal.pone.0068318 (PMC3707916; doi:10.1371/journal.pone.0068318)
Supplement: Table S4 — Proteins identified from the differential 2D DIGE analysis (pH 4–7) after WNV infection. (DOCX) [file pone.0068318.s006.docx]

**Table S4:** Proteins identified from the differential 2D DIGE analysis (pH 4-7) after WNV infection.

| **Accession number**  **(Swissprot)** | **Protein name** | **Molecular**  **weight (kDa)** | ***pI*** | **Spot**  **ID** | **Number of MS/MS**  **peptide sequences** | **Sequence**  **Coverage (%)** | **Mascot**  **score** | **WNV-E /mock** | |
| --- | --- | --- | --- | --- | --- | --- | --- | --- | --- |
|  |  |  |  |  |  |  |  | **Average volume ratio** | **Student t-test**  **(*p* value)** |
| **Host proteins** |  |  |  |  |  |  |  |  |  |
| Cytokeleton |  |  |  |  |  |  |  |  |  |
| ACTA_MOUSE | Actin, aortic smooth muscle [Mus musculus] | 42.38 | 5.23 | 951 | 2 | 5.6 | 91 | 1.6 | 5.91e-06 |
| DCTN1_MOUSE | Dynactin subunit 1 [Mus musculus] | 142.27 | 5.66 | 285 | 16 | 14.4 | 320 | 1.8 | 1.23e-04 |
|  |  |  |  | 283 | 11 | 10.6 | 318 | 1.9 | 5.64e-06 |
| DYN1_MOUSE | Dynamin-1 [Mus musculus] | 98.14 | 7.61 | 511 | 15 | 18.7 | 362 | 1.7 | 4.93e-05 |
|  |  |  |  | 502 | 15 | 17.5 | 311 | 1.4 | 3.59e-05 |
|  |  |  |  | 491 | 15 | 16.7 | 319 | 1.6 | 1.30e-06 |
| SPTA2_MOUSE | Spectrin alpha chain, brain [Mus musculus] | 285.22 | 5.20 | 541 | 7 | 3.0 | 154 | 1.6 | 0.005 |
|  |  |  |  | 101 | 11 | 4.8 | 190 | 1.7 | 1.14e-04 |
|  |  |  |  | 100 | 2 | 0.9 | 115 | 1.5 | 1.18e-04 |
| Host response/protein folding | |  |  |  |  |  |  |  |  |
| ALBU_MOUSE | Serum albumin [Mus musculus] | 70.70 | 5.75 | 765 | 13 | 21.5 | 436 | -1.8 | 0.022 |
|  |  |  |  | 618 | 26 | 48.8 | 2101 | -1.4 | 0.003 |
|  |  |  |  | 617 | 23 | 37.2 | 1841 | -1.3 | 0.002 |
| HS90B_MOUSE | Heat shock protein HSP 90-beta [Mus musculus] | 83.57 | 4.97 | 642 | 3 | 5.4 | 171 | 1.3 | 0.007 |
| TCPG_MOUSE | T-complex protein 1 subunit gamma [Mus musculus] | 61.16 | 6.28 | 506 | 2 | 3.7 | 43 | 1.4 | 1.95e-04 |
| Metabolic/biosynthetic process | |  |  |  |  |  |  |  |  |
| ODP2_MOUSE | Dihydrolipoyllysine-residue acetyltransferase component of pyruvate dehydrogenase complex, mitochondrial [Mus musculus] | 68.47 | 8.81 | 679 | 7 | 13.2 | 153 | -2.0 | 2.57e-05 |
| PPME1_MOUSE | Protein phosphatase methylesterase 1 [Mus musculus] | 42.63 | 5.67 | 890 | 1 | 3.1 | 66 | 1.5 | 1.02e-05 |
| Signal transduction | |  |  |  |  |  |  |  |  |
| GNAO_MOUSE | Guanine nucleotide-binding protein G(o) subunit alpha [Mus musculus] | 40.63 | 5.34 | 1007 | 4 | 11.9 | 83 | 1.3 | 6.06e-04 |
| Not identified |  |  |  |  |  |  |  |  |  |
|  | *n.i.* |  |  | 1233 |  |  |  | -1.3 | 0.002 |
|  | *n.i.* |  |  | 1162 |  |  |  | 1.3 | 0.022 |

The proteins were identified by mass spectrometry following in-gel trypsin digestion. The spot numbers correspond to the same numbers as indicated on Figure S1. The identities of the spots, their SwissProt accession numbers, and the theoretical molecular masses and *pI* values as well as the number of peptide sequences, the corresponding percent sequence coverage, and the Mascot score are listed for MS/MS analysis. Protein scores greater than 34 were considered as significant (*p< 0.05*). Paired average volume ratio and *p* values (Student t-test) between WNV-E and mock groups were defined using Progenesis Samespot software. *n.i*., no identification.
