# Supplementary material for: Altered Protein Networks and Cellular Pathways in Severe West Nile Disease in Mice
Source: PLoS One. 2013 Jul 10;8(7):e68318. doi: 10.1371/journal.pone.0068318 (PMC3707916; doi:10.1371/journal.pone.0068318)
Supplement: Table S5 — Set of proteins identified by iTRAQ labeling and tandem mass spectrometry as differentially expressed between mock-, early- and late WNV-infected samples, indicating fold-changes and p-values in each comparison, and GO subcellular location and biological function. (DOC) [file pone.0068318.s007.doc]

**Table S5.** Dataset of proteins identified by iTRAQ labeling and tandem mass spectrometry as differentially expressed between mock-, early- and late WNV-infected samples, indicating fold-changes and p-values in each comparison, subcellular location and GO biological function.

| **Protein name** | **UniprotKB** | | **Gene name** | **Early vs Mock** | | **Late vs Mock** | | **Late vs Early** | |  |  |
| --- | --- | --- | --- | --- | --- | --- | --- | --- | --- | --- | --- |
|  | **Accession n°** | **Entry name** |  | **p-value** | **fold-change** | **p-value** | **fold-change^#^** | **p-value** | **fold-change^#^** | **Subcellular location** | **Function** |
| ***Proteins deregulated at early and late time-points**** | | |  |  |  |  |  |  |  |  |  |
| *down/down* |  |  |  |  |  |  |  |  |  |  |  |
| Peptidyl-prolyl cis-trans isomerase D | Q9CR16 | PPID_MOUSE | Ppid | 4,47E-02 | -10,00 | 4,47E-02 | -10,00 | 4,61E-02 | -10,00 | Cytoplasm | Protein folding |
| Inactive hydroxysteroid dehydrogenase-like protein 1 | Q8BTX9 | HSDL1_MOUSE | Hsdl1 | 3,21E-03 | -10,00 | 1,53E-02 | -10,00 | 7,26E-04 | 10,00 | Mitochondrion | Redox activity |
| Cytoplasmic FMR1-interacting protein 2 | Q5SQX6 | CYFP2_MOUSE | Cyfip2 | 4,46E-02 | -10,00 | 4,46E-02 | -10,00 |  |  | Cytoplasm | Apoptosis |
| Proline-, glutamic acid- and leucine-rich protein 1 | Q9DBD5 | PELP1_MOUSE | Pelp1 | 4,68E-02 | -10,00 | 4,68E-02 | -10,00 |  |  | Nucleus | Transcription |
| Protein TANC2 | A2A690 | TANC2_MOUSE | Tanc2 | 4,54E-02 | -10,00 | 4,54E-02 | -10,00 |  |  | Nucleus | Transcription |
| Suppressor of G2 allele of SKP1 homolog | Q9CX34 | SUGT1_MOUSE | Sugt1 | 4,64E-02 | -10,00 | 4,64E-02 | -10,00 |  |  | Nucleus | Binding |
| Tether containing UBX domain for GLUT4 | Q8VBT9 | ASPC1_MOUSE | Aspscr1 | 4,37E-02 | -10,00 | 4,37E-02 | -10,00 |  |  | Cytoplasm | Transport |
| Casein kinase II subunit alpha | Q60737 | CSK21_MOUSE | Csnk2a1 | 5,69E-03 | -9,09 | 1,87E-02 | -1,45 | 1,58E-03 | 9,46 | Cytoplasm | Transcription |
| Vacuolar protein sorting-associated protein 35 | Q9EQH3 | VPS35_MOUSE | Vps35 | 3,20E-02 | -2,00 | 2,12E-02 | -10,00 |  |  | Cytoplasm | Transport |
| Isoform 3 of E3 ubiquitin-protein ligase HUWE1 | Q7TMY8-3 | HUWE1_MOUSE | Huwe1 | 4,41E-02 | -1,85 | 4,41E-02 | -2,50 |  |  | Nucleus | Transcription |
| Importin-7 | Q9EPL8 | IPO7_MOUSE | Ipo7 | 4,24E-02 | -1,56 | 2,41E-02 | -2,04 |  |  | Nucleus | Transport |
| pericentriolar material 1 | E9QMG0 | E9QMG0_MOUSE | Pcm1 | 4,69E-02 | -1,45 | 5,38E-03 | -2,13 | 7,74E-03 | -1,69 | Cytoplasm | Other |
| *down/up* |  |  |  |  |  |  |  |  |  |  |  |
| SMT3 suppressor of mif two 3 homolog 3 (S. cerevisiae) | G3UZA7 | G3UZA7_MOUSE | Sumo3 | 4,32E-02 | -10,00 | 2,49E-02 | 6,08 | 2,36E-02 | 10,00 | Nucleus | Protein modification |
| Ubiquitin-conjugating enzyme E2 variant 1 | Q9CZY3 | UB2V1_MOUSE | Ube2v1 | 5,32E-03 | -10,00 | 4,81E-04 | 10,00 | 1,79E-03 | 8,55 | Nucleus | Transcription |
| Carbamoyl-phphate synthetase 2, aspartate transcarbamylase, and dihydroorotase | B2RQC6 | B2RQC6_MOUSE | Cad | 3,89E-02 | -10,00 | 1,67E-02 | 10,00 | 1,67E-02 | 10,00 | Cytoplasm | Metabolic |
| Serotransferrin | Q921I1 | TRFE_MOUSE | Tf | 2,97E-02 | -5,00 | 2,54E-06 | 3,53 | 2,31E-07 | 1,9 | Extracellular Space | Transcription |
| Beta-globin | A8DUK4 | A8DUK4_MOUSE | Hbb-b1 | 1,72E-02 | -3,03 | 7,55E-03 | 1,85 | 5,34E-04 | 3,84 | Cytoplasm | Transport |
| ATP synthase subunit beta, mitochondrial | P56480 | ATPB_MOUSE | Atp5b | 3,63E-02 | -1,89 | 2,48E-03 | 3,73 |  |  | Mitochondrion. | Transport |
| Serum albumin | P07724 | ALBU_MOUSE | Alb | 2,00E-03 | -1,30 | 8,44E-09 | 2,23 | 6,66E-12 | 10,00 | Extracellular Space | Host response |
| *up/up* |  |  |  |  |  |  |  |  |  |  |  |
| Aconites hydratase, mitochondrial | Q99KI0 | ACON_MOUSE | Aco2 | 2,35E-02 | 1,45 | 1,31E-02 | 1,45 |  |  | Mitochondrion. | Metabolic |
| Synapsin-1 | O88935 | SYN1_MOUSE | Syn1 | 6,85E-03 | 1,67 | 6,26E-03 | 1,67 |  |  | Golgi apparatus | Nervous System |
| Clathrin, heavy polypeptide (Hc) | Q5SXR6 | Q5SXR6_MOUSE | Cltc | 8,44E-03 | 1,75 | 2,64E-04 | 2,33 |  |  | Membrane | Transport |
| Glial fibrillary acidic protein | P03995 | GFAP_MOUSE | Gfap | 1,66E-02 | 1,96 | 1,26E-04 | 1,40 |  |  | Cytoplasm | Nervous system |
| Spermidine synthase | Q64674 | SPEE_MOUSE | Srm | 3,31E-02 | 1,96 | 3,17E-02 | 2,47 |  |  | Cytoplasm | Metabolic |
| heterogeneous nuclear ribonucleoprotein A2/B1 | F6U106 | F6U106_MOUSE | Hnrnpa2b1 | 1,45E-02 | 2,09 | 1,38E-02 | 3,25 |  |  | Nucleus | Binding |
| Pnp protein | Q543K9 | Q543K9_MOUSE | Pnp | 1,56E-03 | 3,08 | 1,39E-03 | 4,92 |  |  | Nucleus | Metabolic |
| TAP binding protein (tapasin) | Q3TCU5 | Q3TCU5_MOUSE | Tapbp | 1,75E-02 | 3,44 | 1,57E-02 | 10,00 | 1,57E-02 | 6,61 | Cytoplasm | Host response |
| Guanine nucleotide-binding protein G(q) subunit alpha | P21279 | GNAQ_MOUSE | Gnaq | 2,46E-02 | 3,44 | 2,48E-02 | 3,28 |  |  | Membrane | Nervous system |
| Haptoglobin | Q61646 | HPT_MOUSE | Hp | 1,85E-03 | 4,09 | 1,45E-03 | 7,73 |  |  | Extracellular Space | Proteolysis |
| G protein-coupled receptor kinase-interactor 1 | Q5F258 | Q5F258_MOUSE | Git1 | 4,28E-02 | 6,03 | 4,35E-02 | 6,37 |  |  | Nucleus | Binding |
| Protein tyrine phphatase, receptor type Z, polypeptide 1 | B9EKR1 | B9EKR1_MOUSE | Ptprz1 | 2,25E-02 | 6,67 | 2,38E-02 | 3,50 |  |  | Membrane | Nervous system |
| Apoptis inhibitor 5 | O35841 | API5_MOUSE | Api5 | 2,63E-02 | 7,05 | 2,57E-02 | 8,71 |  |  | Cytoplasm | Apoptosis |
| myosin XVIIIA | E9QAX2 | E9QAX2_MOUSE | Myo18a | 3,78E-02 | 7,53 | 3,60E-02 | 10,00 |  |  | Cytoplasm | Other |
| Actin-related protein 2/3 complex subunit 5 | Q9CPW4 | ARPC5_MOUSE | Arpc5 | 2,42E-02 | 8,02 | 2,39E-02 | 9,38 |  |  | Cytoskeleton | Cytoskeleton organization |
| Signal transducer and activator of transcription 1 | P42225 | STAT1_MOUSE | Stat1 | 1,91E-02 | 10,00 | 1,92E-02 | 10,00 | 3,85E-02 | 10,00 | Nucleus | Transcription |
| Putative ATP-dependent Clp protease proteolytic subunit, mitochondrial | O88696 | CLPP_MOUSE | Clpp | 1,59E-02 | 10,00 | 1,69E-02 | 9,46 |  |  | Mitochondrion. | Proteolysis |
| Basigin | P18572 | BASI_MOUSE | Bsg | 2,61E-02 | 10,00 | 2,67E-02 | 10,00 |  |  | Membrane | Transport |
| ubiquitin-conjugating enzyme E2M | F6YXS3 | F6YXS3_MOUSE | Ube2m | 2,62E-02 | 10,00 | 2,64E-02 | 10,00 |  |  | Cytoplasm | enzyme |
| Signal transducer and activator of transcription 2 | Q9WVL2 | STAT2_MOUSE | Stat2 | 1,49E-02 | 10,00 | 1,48E-02 | 10,00 |  |  | Nucleus | Transcription |
| Opioid binding protein/cell adhesion molecule-like | Q6DFY2 | Q6DFY2_MOUSE | Opcml | 1,71E-02 | 10,00 | 1,68E-02 | 10,00 |  |  | Membrane | transmembrane receptor |
| Methionine aminopeptidase 2 | O08663 | AMPM2_MOUSE | Metap2 | 1,99E-02 | 10,00 | 1,98E-02 | 10,00 |  |  | Cytoplasm | Proteolysis |
| 26S proteasome non-ATPase regulatory subunit 2 | Q8VDM4 | PSMD2_MOUSE | Psmd2 | 2,01E-02 | 10,00 | 2,01E-02 | 10,00 |  |  | Cytoplasm | Metabolic |
| WD repeat domain 72 | D3YYM4 | D3YYM4_MOUSE | Wdr72 | 1,34E-02 | 10,00 | 1,33E-02 | 10,00 |  |  | unknown | Other |
| Hepatocyte cell adhesion molecule | Q640R3 | HECAM_MOUSE | Hepacam | 1,60E-02 | 10,00 | 1,66E-02 | 10,00 |  |  | Cytoplasm | cell division |
| Calpain-9 | Q9D805 | CAN9_MOUSE | Capn9 | 1,36E-02 | 10,00 | 1,35E-02 | 10,00 |  |  | Cytoplasm | Proteolysis |
| Aldehyde dehydrogenase, mitochondrial | P47738 | ALDH2_MOUSE | Aldh2 | 1,34E-02 | 10,00 | 1,35E-02 | 10,00 |  |  | Mitochondrion | Metabolic |
| Cell differentiation protein RCD1 homolog | Q9JKY0 | RCD1_MOUSE | Rqcd1 | 1,70E-02 | 10,00 | 1,70E-02 | 10,00 |  |  | Cytoplasm | Transcription |
| Transformation/transcription domain-associated protein | E9QLK7 | E9QLK7_MOUSE | Trrap | 1,52E-02 | 10,00 | 1,58E-02 | 10,00 |  |  | Nucleus | Transcription |
| *up/down* |  |  |  |  |  |  |  |  |  |  |  |
| Vacuolar protein sorting 29 homolog (S. cerevisiae) | D3Z645 | D3Z645_MOUSE | Vps29 | 2,01E-02 | 9,46 | 4,38E-02 | -10,00 |  |  | Cytoplasm | Transport |
| ATP-binding cassette sub-family F member 3 | Q8K268 | ABCF3_MOUSE | Abcf3 | 1,69E-02 | 10,00 | 4,25E-02 | -10,00 | 4,47E-02 | -10,00 | unknown | Transport |
| ***Proteins deregulated at the late time-point**** |  |  |  |  |  |  |  |  |  |  |  |
| *down* |  |  |  |  |  |  |  |  |  |  |  |
| Alpha-soluble NSF attachment protein | Q9DB05 | SNAA_MOUSE | Napa |  |  | 2,64E-02 | -10,00 | 2,61E-02 | -10,00 | Cytoplasm | Nervous system |
| Chloride channel 6 | A2A7F6 | A2A7F6_MOUSE | Clcn6 |  |  | 3,14E-02 | -5,26 | 3,40E-02 | -6,25 | Membrane | Ion channel |
| cAMP-dependent protein kinase catalytic subunit beta | P68181 | KAPCB_MOUSE | Prkacb |  |  | 6,02E-03 | -2,27 | 6,96E-03 | -3,23 | Cytoplasm | Binding |
| 40S ribomal protein S15a | P62245 | RS15A_MOUSE | Rps15a |  |  | 3,26E-02 | -2,13 |  |  | Cytoplasm | Translation |
| Fatty acid synthase | P19096 | FAS_MOUSE | Fasn |  |  | 7,61E-03 | -2,00 | 8,73E-03 | -4,00 | Melanosome. | Metabolic |
| Prolow-density lipoprotein receptor-related protein 1 | Q91ZX7 | LRP1_MOUSE | Lrp1 |  |  | 2,57E-02 | -1,96 |  |  | Membrane | Transmembrane receptor |
| Amyloid beta A4 protein | P12023 | A4_MOUSE | App |  |  | 1,62E-02 | -1,79 | 2,29E-02 | -10,00 | Membrane | Nervous system |
| *up* |  |  |  |  |  |  |  |  |  |  |  |
| MKIAA0968 protein (Fragment) | Q80TN1 | Q80TN1_MOUSE | Camk2a |  |  | 3,53E-04 | 1,31 | 2,04E-03 | 2,01 | Cytoplasm | Nervous system |
| 60S ribomal protein L7 | P14148 | RL7_MOUSE | Rpl7 |  |  | 1,47E-02 | 1,31 |  |  | Cytoplasm | Transcription |
| Superoxide dismutase [Cu-Zn] | P08228 | SODC_MOUSE | Sod1 |  |  | 1,84E-02 | 1,32 |  |  | Cytoplasm | Host response |
| Alpha-enolase | P17182 | ENOA_MOUSE | Eno1 |  |  | 1,76E-02 | 1,32 |  |  | Cytoplasm | Metabolic |
| ADP/ATP translocase 2 | P51881 | ADT2_MOUSE | Slc25a5 |  |  | 1,11E-02 | 1,34 |  |  | Mitochondrion | Transport |
| Vimentin | P20152 | VIME_MOUSE | Vim |  |  | 1,27E-03 | 1,39 | 8,02E-04 | 1,50 | Cytoplasm | Nervous system |
| Sodium/potassium-transporting ATPase subunit beta-1 | P14094 | AT1B1_MOUSE | Atp1b1 |  |  | 1,10E-03 | 1,50 |  |  | Membrane | Transport |
| L-lactate dehydrogenase A chain | P06151 | LDHA_MOUSE | Ldha |  |  | 2,58E-03 | 1,61 |  |  | Cytoplasm | Metabolic |
| Dynamin-1 (EC 3.6.5.5) | P39053 | DYN1_MOUSE | Dnm1 |  |  | 1,23E-02 | 1,64 |  |  | Cytoskeleton | Cytoskeleton organization |
| Syntaxin-binding protein 1 | O08599 | STXB1_MOUSE | Stxbp1 |  |  | 3,08E-05 | 1,64 |  |  | Cytoplasm | Nervous system |
| Serine/threonine-protein phphatase 2A 65 kDa regulatory subunit A alpha isoform | Q76MZ3 | 2AAA_MOUSE | Ppp2r1a |  |  | 3,52E-02 | 1,69 |  |  | Cytoplasm | Apoptosis |
| Gluce-6-phphate isomerase | P06745 | G6PI_MOUSE | Gpi |  |  | 2,56E-04 | 1,75 |  |  | Extracellular Space | Metabolic |
| Fructose-bisphosphate aldolase | A6ZI44 | A6ZI44_MOUSE | Aldoa |  |  | 2,47E-02 | 1,82 |  |  | Cytoplasm | Metabolic |
| Creatine kinase B-type | Q04447 | KCRB_MOUSE | Ckb |  |  | 2,84E-04 | 1,87 |  |  | Cytoplasm | Binding |
| Moesin | P26041 | MOES_MOUSE | Msn |  |  | 2,56E-02 | 1,91 |  |  | Membrane | Cytoskeleton organization |
| Uncharacterized protein | Q8BPD5 | Q8BPD5_MOUSE | Apoa1 |  |  | 7,69E-03 | 1,96 |  |  | Extracellular Space | Transport |
| Sodium/potassium-transporting ATPase subunit alpha-1 | Q8VDN2 | AT1A1_MOUSE | Atp1a1 |  |  | 2,78E-02 | 2,00 |  |  | Membrane | Transport |
| Hemopexin | Q91X72 | HEMO_MOUSE | Hpx |  |  | 2,89E-02 | 2,05 |  |  | Extracellular Space | Transport |
| Spectrin beta chain, brain 1 | Q62261 | SPTB2_MOUSE | Sptbn1 |  |  | 9,03E-03 | 2,33 |  |  | Membrane | Transport |
| Myelin basic protein (Fragment) | F6VME3 | F6VME3_MOUSE | Mbp |  |  | 7,51E-03 | 2,88 |  |  | Extracellular Space | Nervous system |
| Spectrin alpha 2 | A3KGU7 | A3KGU7_MOUSE | Spna2 |  |  | 8,14E-04 | 3,34 |  |  | Membrane | Binding |
| Plasma membrane calcium-transporting ATPase 2 | F8WHB1 | F8WHB1_MOUSE | Atp2b2 |  |  | 1,52E-03 | 3,40 |  |  | Membrane | Transport |
| Catenin beta-1 | Q02248 | CTNB1_MOUSE | Ctnnb1 |  |  | 3,35E-02 | 3,70 | 3,42E-02 | 2,58 | Nucleus | Transcription |
| H-2 class I histocompatibility antigen, D-B alpha chain | E9QQ06 | E9QQ06_MOUSE | H2-D1 |  |  | 4,18E-02 | 6,61 |  |  | Membrane | Host response |
| T-complex protein 1 subunit epsilon | P80316 | TCPE_MOUSE | Cct5 |  |  | 2,43E-02 | 7,11 |  |  | Cytoplasm | Protein folding |
| Metal transporter CNNM3 | Q32NY4 | CNNM3_MOUSE | Cnnm3 |  |  | 1,99E-02 | 10,00 | 1,99E-02 | 10,00 | Membrane | Transport |
| ***Proteins deregulated at the early time-point**** | |  |  |  |  |  |  |  |  |  |  |
| *down* |  |  |  |  |  |  |  |  |  |  |  |
| F-box/LRR-repeat protein 16 | A2RT62 | FXL16_MOUSE | Fbxl16 | 4,45E-02 | -10,00 |  |  | 1,68E-02 | 10,00 | unknown | Other |
| 40S ribomal protein S21 | Q9CQR2 | RS21_MOUSE | Rps21 | 2,39E-02 | -10,00 |  |  | 1,26E-02 | 10,00 | Cytoplasm | Translation |
| Erythrocyte membrane protein band 4.2 | P49222 | EPB42_MOUSE | Epb42 | 4,37E-02 | -10,00 |  |  | 1,63E-02 | 10,00 | Plasma Membrane | Cytoskeleton organization |
| 26S protease regulatory subunit 6A | O88685 | PRS6A_MOUSE | Psmc3 | 2,63E-02 | -10,00 |  |  | 1,33E-02 | 10,00 | Nucleus | Transcription regulator |
| Ras-related GTP-binding protein C | Q99K70 | RRAGC_MOUSE | Rragc | 3,75E-02 | -10,00 |  |  | 1,60E-02 | 10,00 | Cytoplasm | Protein modification |
| Calcium/calmodulin-dependent protein kinase IV | Q8BGR3 | Q8BGR3_MOUSE | Camk4 | 2,31E-02 | -10,00 |  |  | 1,25E-02 | 10,00 | Nucleus | Transport |
| Cyclin-dependent kinase 5 | P49615 | CDK5_MOUSE | Cdk5 | 3,99E-02 | -10,00 |  |  | 1,37E-02 | 4,74 | Nucleus | Nervous System |
| Enoyl-CoA hydratase, mitochondrial | Q8BH95 | ECHM_MOUSE | Echs1 | 4,62E-02 | -2,22 |  |  |  |  | Mitochondrion. | Metabolic |
| ADP-ribylation factor-like protein 8B | Q9CQW2 | ARL8B_MOUSE | Arl8b | 4,34E-02 | -1,79 |  |  | 2,43E-02 | 1,50 | Plasma Membrane | Cell division |
| Neutral alpha-glucidase AB | Q8BHN3 | GANAB_MOUSE | Ganab | 2,67E-02 | -1,79 |  |  | 2,70E-02 | 1,51 | Endoplasmic reticulum | Metabolic |
| Wiskott-Aldrich syndrome protein family member 1 | Q8R5H6 | WASF1_MOUSE | Wasf1 | 3,65E-02 | -1,54 |  |  | 1,65E-02 | 1,47 | Cytoskeleton | Cytoskeleton organization |
| Tripartite motif-containing protein 3 | Q9R1R2 | TRIM3_MOUSE | Trim3 | 4,21E-02 | -1,45 |  |  |  |  | Cytoplasm | Transport |
| centrosomal protein 290kDa | E9Q9M0 | E9Q9M0_MOUSE | Cep290 | 2,78E-02 | -1,43 |  |  |  |  | Cytoplasm | Transcription regulator |
| Ras-related protein Rab-2A | P53994 | RAB2A_MOUSE | Rab2a | 2,51E-02 | -1,35 |  |  |  |  | Endoplasmic reticulum | Transport |
| RIKEN cDNA 2310035C23, isoform CRA_a | G3X9J4 | G3X9J4_MOUSE | 2310035C23Rik | 2,92E-02 | -1,35 |  |  |  |  | unknown | Binding |
| V-type proton ATPase subunit H | Q8BVE3 | VATH_MOUSE | Atp6v1h | 8,71E-03 | -1,35 |  |  |  |  | Cytoplasm | Transport |
| Src substrate cortactin | Q60598 | SRC8_MOUSE | Cttn | 1,73E-03 | -1,30 |  |  | 4,58E-04 | 1,34 | Cytoskeleton | Cytoskeleton organization |
| *up* |  |  |  |  |  |  |  |  |  |  |  |
| Microtubule-associated protein 1B | P14873 | MAP1B_MOUSE | Map1b | 2,47E-03 | 1,34 |  |  | 1,53E-03 | -2,63 | Cytoskeleton | Nervous system |
| T-complex protein 1 subunit gamma | P80318 | TCPG_MOUSE | Cct3 | 1,95E-04 | 1,40 |  |  |  |  | Cytoplasm | Protein folding |
| Phphoglycerate kinase 1 | P09411 | PGK1_MOUSE | Pgk1 | 5,72E-04 | 1,42 |  |  |  |  | Cytoplasm | Metabolic |
| Dihydropyrimidinase-related protein 2 | O08553 | DPYL2_MOUSE | Dpysl2 | 2,16E-04 | 1,46 |  |  | 2,78E-06 | -1,3 | Cytoplasm | Nervous System |
| Microtubule-associated protein 2 | P20357 | MAP2_MOUSE | Map2 | 6,66E-06 | 1,47 |  |  | 1,47E-03 | -1,33 | Cytoskeleton | Nervous System |
| High mobility group protein B1 | P63158 | HMGB1_MOUSE | Hmgb1 | 2,22E-02 | 1,50 |  |  |  |  | Nucleus | Transcription regulator |
| neural cell adhesion molecule 1 | F7C5V8 | F7C5V8_MOUSE | Ncam1 | 2,52E-03 | 1,51 |  |  | 1,58E-03 | -1,64 | Plasma Membrane | Nervous System |
| Peroxisomal multifunctional enzyme type 2 | P51660 | DHB4_MOUSE | Hsd17b4 | 2,35E-02 | 1,53 |  |  | 3,15E-02 | -1,79 | Peroxisome. | Metabolic |
| Glyceraldehyde-3-phphate dehydrogenase | E9PX42 | E9PX42_MOUSE | Gm2606 | 4,32E-02 | 1,54 |  |  |  |  | Plasma Membrane | Metabolic |
| T-complex protein 1 subunit delta | P80315 | TCPD_MOUSE | Cct4 | 7,55E-03 | 1,69 |  |  |  |  | Cytoplasm | Protein folding |
| Neuromodulin | P06837 | NEUM_MOUSE | Gap43 | 1,39E-02 | 1,77 |  |  |  |  | Plasma Membrane | Nervous System |
| MARCKS-related protein | P28667 | MRP_MOUSE | Marcksl1 | 2,69E-02 | 2,09 |  |  |  |  | Cytoplasm | Cell division |
| Serine/threonine-protein kinase ULK3 | Q3U3Q1 | ULK3_MOUSE | Ulk3 | 2,01E-02 | 10,00 |  |  |  |  | Cytoplasm | Protein modification |
| Cat eye syndrome critical region protein 6 homolog | Q99MX7 | CECR6_MOUSE | Cecr6 | 1,95E-02 | 10,00 |  |  |  |  | Nucleus | Transcription regulator |
| Arginyl-tRNA synthetase, cytoplasmic | Q9D0I9 | SYRC_MOUSE | Rars | 2,00E-02 | 10,00 |  |  |  |  | Cytoplasm | Metabolic |
| Protein prune homolog | Q8BIW1 | PRUNE_MOUSE | Prune | 2,01E-02 | 10,00 |  |  |  |  | Nucleus | Binding |
| ***Proteins deregulated only between late and early time-points*** | | |  |  |  |  |  |  |  |  |  |
| *down* |  |  |  |  |  |  |  |  |  |  |  |
| Crmp1 protein | Q6P1J1 | Q6P1J1_MOUSE | Crmp1 |  |  |  |  | 8,03E-03 | -1,56 | unknown | Nervous system |
| Serine/threonine-protein kinase A-Raf | P04627 | ARAF_MOUSE | Araf |  |  |  |  | 2,76E-02 | -1,32 | unknown | Signaling |
| up |  |  |  |  |  |  |  |  |  |  |  |
| Carbonyl reductase [NADPH] 1 | P48758 | CBR1_MOUSE | Cbr1 |  |  |  |  | 2,17E-03 | 1,42 | Cytoplasm | Bio process |
| Heat shock-related 70 kDa protein 2 | P17156 | HSP72_MOUSE | Hspa2 |  |  |  |  | 2,87E-02 | 1,46 | unknown | Host response |
| Vacuolar protein sorting-associated protein 45 | P97390 | VPS45_MOUSE | Vps45 |  |  |  |  | 3,21E-02 | 1,47 | Golgi | Transport |
| Alpha globin 1 | Q91VB8 | Q91VB8_MOUSE | Hba-a1 |  |  |  |  | 1,70E-02 | 2,54 | unknown | Transport |
| Ran-binding protein 3 | Q9CT10 | RANB3_MOUSE | Ranbp3 |  |  |  |  | 2,68E-02 | 5,86 | Nucleus | Transport |
| Alpha-2-HS-glycoprotein | P29699 | FETUA_MOUSE | Ahsg |  |  |  |  | 1,70E-02 | 7,80 | Extracellular Space | Host response |
|  |  |  |  |  |  |  |  |  |  |  |  |

* as compared to mock-infected samples; # absolute fold-change equal or upper than 10 were indicated as 10.
