# Supplementary material for: Altered Protein Networks and Cellular Pathways in Severe West Nile Disease in Mice
Source: PLoS One. 2013 Jul 10;8(7):e68318. doi: 10.1371/journal.pone.0068318 (PMC3707916; doi:10.1371/journal.pone.0068318)
Supplement: Table S6 — Ingenuity canonical pathways showing a significant association using the set of proteins that are differentially expressed between early- and mock-WNV infected samples [−Log(p-value) >2.0]. (DOCX) [file pone.0068318.s008.docx]

**Table S6: Ingenuity Canonical Pathways showing a significant association using the dataset of proteins differentially expressed between early- and Mock-WNV infected samples [-Log(p-value) >2.0].**

| **Canonical Pathways** | **-Log(p-value)** | **Molecules** |
| --- | --- | --- |
| Clathrin-mediated Endocytosis Signaling | 7.17 | HSPA8, DNM1, ALB, TF, ACTB, ACTA2, ARPC5, CLTC, CSNK2A1, ACTG2, CTTN |
| Huntington's Disease Signaling | 4.6 | HSPA8, DNM1, ATP5B, CDK5, CLTC, GNAQ, DCTN1, CAPN9 |
| Signaling by Rho Family GTPases | 4.4 | ACTB, ACTA2, ARPC5, GNAO1, GNAQ, GFAP, WASF1, ACTG2 |
| RhoGDI Signaling | 4.31 | ACTB, ACTA2, ARPC5, GNAO1, GNAQ, WASF1, ACTG2 |
| β-alanine Metabolism | 4.04 | DPYSL2, ALDH2, SRM, ECHS1 |
| Regulation of Actin-based Motility by Rho | 4.0 | ACTB, ACTA2, ARPC5, WASF1, ACTG2 |
| Virus Entry via Endocytic Pathways | 3.86 | DNM1, ACTB, ACTA2, CLTC, ACTG2 |
| Actin Cytoskeleton Signaling | 3.72 | CYFIP2, ACTB, ACTA2, ARPC5, WASF1, ACTG2, GIT1 (includes EG:216963) |
| RhoA Signaling | 3.43 | ACTB, ACTA2, ARPC5, WASF1, ACTG2 |
| Protein Ubiquitination Pathway | 3.28 | HSPA8, UBE2M, HSP90AB1, PSMD2, UBE2V1, SUGT1, PSMC3 |
| Caveolar-mediated Endocytosis Signaling | 3.13 | ALB, ACTB, ACTA2, ACTG2 |
| Integrin Signaling | 3.09 | RALA, ACTB, ACTA2, ARPC5, CAPN9, ACTG2, GIT1 (includes EG:216963), CTTN |
| Cellular Effects of Sildenafil (Viagra) | 3.04 | CAMK4, ACTB, ACTA2, GNAQ, ACTG2 |
| FAK Signaling | 2.87 | ACTB, ACTA2, CAPN9, ACTG2 |
| Mechanisms of Viral Exit from Host Cells | 2.86 | ACTB, ACTA2, ACTG2 |
| Fcγ Receptor-mediated Phagocytosis in Macrophages and Monocytes | 2.76 | ACTB, ACTA2, ARPC5, ACTG2 |
| MSP-RON Signaling Pathway | 2.68 | ACTB, ACTA2, ACTG2 |
| Amyloid Processing | 2.55 | CDK5, CSNK2A1, CAPN9 |
| Corticotropin Releasing Hormone Signaling | 2.49 | CAMK4, ARPC5, GNAO1, GNAQ |
| Butanoate Metabolism | 2.37 | ALDH2, ECHS1, HSD17B4 |
| Fatty Acid Elongation in Mitochondria | 2.36 | ECHS1, HSD17B4 |
| Lysine Degradation | 2.35 | ALDH2, ECHS1, HSD17B4 |
| Hypoxia Signaling in the Cardiovascular System | 2.27 | UBE2M, HSP90AB1, UBE2V1 |
| Valine, Leucine and Isoleucine Degradation | 2.25 | ALDH2, ECHS1, HSD17B4 |
| eNOS Signaling | 2.23 | HSPA8, CAMK4, HSP90AB1, GNAQ |
| JAK/Stat Signaling | 2.22 | GNAQ, STAT2, STAT1 |
| Agrin Interactions at Neuromuscular Junction | 2.2 | ACTB, ACTA2, ACTG2, CTTN |
| Melatonin Signaling | 2.13 | CAMK4, GNAO1, GNAQ |
| Arginine and Proline Metabolism | 2.12 | ALDH2, SRM, RARS |
| Role of JAK1, JAK2 and TYK2 in Interferon Signaling | 2.11 | STAT2, STAT1 |
