# Supplementary material for: Altered Protein Networks and Cellular Pathways in Severe West Nile Disease in Mice
Source: PLoS One. 2013 Jul 10;8(7):e68318. doi: 10.1371/journal.pone.0068318 (PMC3707916; doi:10.1371/journal.pone.0068318)
Supplement: Table S7 — Ingenuity canonical pathways showing a significant association using the set of proteins that are differentially expressed between late- and mock-WNV infected samples [−Log(p-value) >2.0]. (DOCX) [file pone.0068318.s009.docx]

**Table S7: Ingenuity Canonical Pathways showing a significant association using the dataset of proteins differentially expressed between late- and Mock-WNV infected samples [-Log(p-value) >2.0].**

| **Canonical Pathways** | **-Log(p-value)** | **Molecules** |
| --- | --- | --- |
| Clathrin-mediated Endocytosis Signaling | 5.98 | HSPA8, DNM1, ALB, TF, APOA1, ACTB, ARPC5, CLTC, CSNK2A1 |
| Glycolysis/Gluconeogenesis | 3.85 | ALDH2, GPI, ENO1, ALDOA, LDHA |
| Amyloid Processing | 3.69 | PRKACB, CSNK2A1, CAPN9, APP |
| Huntington's Disease Signaling | 3.62 | HSPA8, DNM1, ATP5B, CLTC, GNAQ, CAPN9, NAPA (includes EG:108124) |
| Protein Ubiquitination Pathway | 3.19 | HSPA8, UBE2M, PSMD2, UBE2V1, SUGT1, HSPA12A, HLA-C |
| LXR/RXR Activation | 3.19 | ALB, HPX, TF, APOA1, FASN |
| Neuroprotective Role of THOP1 in Alzheimer's Disease | 2.81 | PRKACB, APP, HLA-C |
| Virus Entry via Endocytic Pathways | 2.75 | DNM1, ACTB, CLTC, HLA-C |
| β-alanine Metabolism | 2.72 | DPYSL2, ALDH2, SRM |
| Tight Junction Signaling | 2.71 | PRKACB, PPP2R1A, ACTB, SPTAN1, CTNNB1 |
| Signaling by Rho Family GTPases | 2.69 | ACTB, ARPC5, GNAQ, VIM, GFAP, MSN |
| Acute Phase Response Signaling | 2.58 | ALB, HPX, HP, TF, APOA1 |
| Wnt/β-catenin Signaling | 2.56 | PPP2R1A, CSNK2A1, GNAQ, CTNNB1, LRP1 (includes EG:16971) |
| PPARα/RXRα Activation | 2.54 | PRKACB, GPD2, APOA1, FASN, GNAQ |
| Sertoli Cell-Sertoli Cell Junction Signaling | 2.45 | PRKACB, SPTBN1, ACTB, SPTAN1, CTNNB1 |
| GNRH Signaling | 2.25 | PRKACB, DNM1, CAMK2A, GNAQ |
| Hypoxia Signaling in the Cardiovascular System | 2.23 | UBE2M, UBE2V1, LDHA |
| JAK/Stat Signaling | 2.18 | GNAQ, STAT2, STAT1 |
| Actin Cytoskeleton Signaling | 2.1 | CYFIP2, ACTB, ARPC5, GIT1 (includes EG:216963), MSN |
| Melatonin Signaling | 2.09 | PRKACB, CAMK2A, GNAQ |
| Role of JAK1, JAK2 and TYK2 in Interferon Signaling | 2.08 | STAT2, STAT1 |
| Arginine and Proline Metabolism | 2.07 | CKB, ALDH2, SRM |
| Caveolar-mediated Endocytosis Signaling | 2.06 | ALB, ACTB, HLA-C |
