# Supplementary material for: Altered Protein Networks and Cellular Pathways in Severe West Nile Disease in Mice
Source: PLoS One. 2013 Jul 10;8(7):e68318. doi: 10.1371/journal.pone.0068318 (PMC3707916; doi:10.1371/journal.pone.0068318)
Supplement: Table S8 — Ingenuity canonical pathways showing a significant association using the dataset of proteins that are differentially expressed between late- and early-WNV infected samples [−Log(p-value) >2.0]. (DOCX) [file pone.0068318.s010.docx]

**Table S8: Ingenuity Canonical Pathways showing a significant association using the dataset of proteins differentially expressed between late- and early-WNV infected samples [-Log(p-value) >2.0].**

| **Canonical Pathways** | **-Log(p-value)** | **Molecules** |
| --- | --- | --- |
| Clathrin-mediated Endocytosis Signaling | 5.17 | HSPA8, DNM1, ALB, TF, ACTB, CSNK2A1, CTTN |
| Amyloid Processing | 4.35 | PRKACB, CDK5, CSNK2A1, APP |
| Semaphorin Signaling in Neurons | 4.32 | DPYSL2, CRMP1, CDK5, DPYSL3 |
| Melatonin Signaling | 3.76 | PRKACB, CAMK4, CAMK2A, ARAF |
| LXR/RXR Activation | 2.91 | ALB, TF, FASN, AHSG |
| Huntington's Disease Signaling | 2.83 | HSPA8, DNM1, CDK5, NAPA (includes EG:108124), HSPA2 |
| eNOS Signaling | 2.79 | PRKACB, HSPA8, CAMK4, HSPA2 |
| Methane Metabolism | 2.71 | CAT, PRDX6 |
| Pantothenate and CoA Biosynthesis | 2.61 | DPYSL2, CRMP1 |
| Protein Ubiquitination Pathway | 2.51 | HSPA8, UBE2V1, PSMC3, HSPA2, HSPA12A |
| Gap Junction Signaling | 2.45 | PRKACB, ACTB, TUBB, CTNNB1 |
| Sertoli Cell-Sertoli Cell Junction Signaling | 2.3 | PRKACB, ACTB, TUBB, CTNNB1 |
| Breast Cancer Regulation by Stathmin1 | 2.18 | PRKACB, CAMK4, CAMK2A, TUBB |
| Synaptic Long Term Potentiation | 2.12 | PRKACB, CAMK4, CAMK2A |
| Neuropathic Pain Signaling In Dorsal Horn Neurons | 2.12 | PRKACB, CAMK4, CAMK2A |
